# CEST MRI and MALDI imaging reveal metabolic alterations in the cervical lymph nodes of EAE mice

**DOI:** 10.1186/s12974-022-02493-z

**Published:** 2022-06-03

**Authors:** Aline M. Thomas, Ethan Yang, Matthew D. Smith, Chengyan Chu, Peter A. Calabresi, Kristine Glunde, Peter C. M. van Zijl, Jeff W. M. Bulte

**Affiliations:** 1 Russell H. Morgan Department of Radiology and Radiological Science, Division of MR Research, Johns Hopkins University School of Medicine, MD 21205 Baltimore, USA; 2 Cellular Imaging Section and Vascular Biology Program, Institute for Cell Engineering, Johns Hopkins University School of Medicine, Baltimore, MD USA; 3 Department of Neurology, Johns Hopkins University School of Medicine, Baltimore, MD USA; 4 Solomon H Snyder Department of Neuroscience, Johns Hopkins University School of Medicine, Baltimore, MD USA; 5 Department of Oncology, Johns Hopkins University School of Medicine, Baltimore, MD USA; 6 Department of Biological Chemistry, Johns Hopkins University School of Medicine, Baltimore, MD USA; 7 F.M. Kirby Research Center for Functional Brain Imaging, Kennedy Krieger Institute, Baltimore, MD USA; 8 Department of Biomedical Engineering, Johns Hopkins University School of Medicine, Baltimore, MD USA; 9 Department of Chemical and Biomolecular Engineering, Johns Hopkins University School of Medicine, Baltimore, MD USA

**Keywords:** Lymph nodes, Multiple sclerosis, CEST MRI, Neuroinflammation

## Abstract

**Background:**

Multiple sclerosis (MS) is a neurodegenerative disease, wherein aberrant immune cells target myelin-ensheathed nerves. Conventional magnetic resonance imaging (MRI) can be performed to monitor damage to the central nervous system that results from previous inflammation; however, these imaging biomarkers are not necessarily indicative of active, progressive stages of the disease. The immune cells responsible for MS are first activated and sensitized to myelin in lymph nodes (LNs). Here, we present a new strategy for monitoring active disease activity in MS, chemical exchange saturation transfer (CEST) MRI of LNs.

**Methods and results:**

We studied the potential utility of conventional (T2-weighted) and CEST MRI to monitor changes in these LNs during disease progression in an experimental autoimmune encephalomyelitis (EAE) model. We found CEST signal changes corresponded temporally with disease activity. CEST signals at the 3.2 ppm frequency during the active stage of EAE correlated significantly with the cellular (flow cytometry) and metabolic (mass spectrometry imaging) composition of the LNs, as well as immune cell infiltration into brain and spinal cord tissue. Correlating primary metabolites as identified by matrix-assisted laser desorption/ionization (MALDI) imaging included alanine, lactate, leucine, malate, and phenylalanine.

**Conclusions:**

Taken together, we demonstrate the utility of CEST MRI signal changes in superficial cervical LNs as a complementary imaging biomarker for monitoring disease activity in MS. CEST MRI biomarkers corresponded to disease activity, correlated with immune activation (surface markers, antigen-stimulated proliferation), and correlated with LN metabolite levels.

**Supplementary Information:**

The online version contains supplementary material available at 10.1186/s12974-022-02493-z.

## Introduction

The pathophysiology responsible for disability in multiple sclerosis (MS) is complex, involving multiple cell types and biomolecules. Activated immune cells infiltrate the central nervous system (CNS) through the blood–brain–barrier (BBB) and then migrate throughout the brain and spinal cord with the aid of the ventricular system [1, 2]. These cells secrete excitotoxic molecules [3] as well as inflammatory cytokines [4] that in turn activate [5, 6] and induce metabolic alterations [7] in local cells. This combination results in neuronal damage and death of myelinating oligodendrocytes supporting them [8, 9]. This cascade ultimately results in focal lesions or atrophy, which can be visualized using traditional anatomical T1- or T2-weighted magnetic resonance imaging (MRI). However, the heterogeneity of the disease amongst patients complicates the interpretation of these images, decreasing their practicality. Their limitations have motivated the exploration of novel noninvasive strategies for monitoring MS.

More recently, imaging research has shifted from developing strategies that monitor CNS damage to those that monitor the immune cells responsible for MS. Preclinically, several in situ labeling methods have been developed to monitor trafficking of immune cells to CNS lesions [10, 11]. However, immunological episodes in MS are initiated in CNS-draining lymph nodes (LNs), wherein immune cells first become activated and sensitized to myelin antigens [12–15]. The myelin antigens responsible for MS progression have been observed in immune cells residing in the CNS-draining LNs of MS patients and its preclinical model, experimental autoimmune encephalomyelitis (EAE) [14, 15]. In EAE these immune cells have been reported to continuously traffic between the CNS and its draining LNs [12, 13].

Metabolic alterations have been reported in immune cells residing in EAE-associated LNs [16], a feature that can be monitored using molecular imaging. Positron emission tomography [17] and hyperpolarized metabolic imaging [18] have already been evaluated in EAE to monitor metabolic changes in the CNS. Still, their widespread use is restricted by the need of specialized equipment to generate the tracers along with their short half-life [19]. Magnetic resonance spectroscopy (MRS) can detect many of these metabolites without the use of a tracer, but its low sensitivity and low spatial resolution limit its application [20, 21].

Chemical exchange saturation transfer (CEST) MRI has emerged as a more sensitive, higher resolution alternative to MRS whose safety and utility has already been demonstrated clinically in MS patients to monitor neurological damage [22, 23]. It is highly sensitive, and can amplify signals from low-concentration metabolites with a factor between 10^2^ and 10^6^ compared to conventional proton MRS [24]. It is applicable for detection of a multitude of biomolecules in the CNS through the presence of exchangeable protons, e.g., amine and hydroxyl groups [20, 25–28]. The recent development of CEST MRI methods to monitor mobile protein [22, 23] and glutamate [29, 30] content in MS patients has expanded the visual information toolkit for monitoring the pathophysiological progression of lesions and cognitive decline. Indeed, we recently demonstrated that CEST MRI signal changes in the CNS occur during the active stage of EAE, 10–19 day post-induction (DPI), and is able to identify regions absent of T2-weighted lesions, where diffuse injuries are frequently observed in this model [31], which can be partially reversed by transplantation of glia-restricted precursor cells [32].

Here, we investigated the utility of CEST MRI of CNS-draining, superficial cervical LNs to monitor MS disease activity and its metabolic changes in an EAE model, using matrix-assisted laser desorption/ionization (MALDI) imaging as adjunct biomarker validation. Of the CNS-draining LNs, superficial cervical LNs were chosen due to their accessibility for complementary clinical evaluation as they are near the skin and palpable. The CEST signal in these LNs at multiple saturation frequencies (ranging from 0.4 to 6.0 ± 0.2 ppm) was compared to multiple clinically routine metrics for monitoring lesion load, CNS atrophy, and disability. These signals were also compared to current metrics for evaluating immune response: antigen-mediated stimulation of peripheral immune cells, cytometric markers, secondary lymphoid organ size, CNS infiltration of immune cells, and metabolic alterations. Correlation to these metrics in the EAE model indicates the potential of CEST MRI of superficial cervical LNs as a complementary metabolic imaging biomarker for predicting disease activity and progression in MS.

## Methods

### CEST MRI phantoms

Metabolites and biomolecules evaluated included d-glucose (Sigma, 50-99-7), L-lactate (Sigma, L7022-5G), L-glutamate (Sigma, G1251-100C), and bovine serum albumin (Sigma, A9647-100G). Samples contained 50 mM of each molecule suspended in agarose gel (0.2% in phosphate buffered saline; Invitrogen 15510-019) and titrated to a pH = 7.3 ± 0.05.

### EAE induction

All animal studies are approved by the Johns Hopkins University Animal Care and Use Committee. Animals received standard husbandry. C57Bl/6 mice (female, 6–10 weeks, Jackson Laboratories, *n* = 5–8) were injected s.c. with 200 μl of emulsion containing incomplete Freund's adjuvant (Sigma F5506) Mycobacterium tuberculosis H37Ra (Difco BD 231141, 5 mg/ml) and myelin oligodendrocyte glycopeptide (MOG_35-55_, Johns Hopkins Synthesis and Sequencing Facility, 0.5 mg/ml). Mice were injected i.p. with 100 μl of pertussis toxin (List Laboratories #180, 300 ng) on the day of induction and 2 days later. Mice were observed daily for signs of paralysis rated using the following score rubric: 0 = asymptomatic; 1 = atonic tail; 2 = partial hind limb paralysis; 3 = paraplegia; 4 = quadriplegia; and 5 = moribund/death. Disease onset was defined as having a paralysis score ≥ 1. Paralysis severity 0–29 DPI was evaluated using three metrics: maximum clinical score, cumulative clinical score (i.e., the summation of scores), and number of severe days (i.e., having a score ≥ 2.5). EAE mice were compared to mice injected without MOG_35-55_ peptide (peripheral inflammation control, *n* = 4) or naïve mice (*n* = 5). These mice were randomly selected into experimental groups and evaluated at random. For post-mortem analysis, tissues were extracted either the day after imaging or 29 DPI.

### In vivo and in vitro MRI

MRI was performed using a horizontal bore spectrometer with a 20 mm surface receiver coil and a 72 mm volume transmit coil (Bruker Biospin 11.7T). To localize LNs, T2-weighted images were acquired with a slice thickness of 0.7 mm. All other images were co-registered with a resolution of 0.14 mm and slice thickness of 2 mm, which permitted imaging of the whole LN based on our observations and reported size [33]. T2-weighted MR images were acquired with an echo time (TE)/repetition time (TR) = 20.5/2000 ms, and a rare factor of 8 with a single average and repetition. CEST MR images were acquired using a continuous-wave sinc-gauss pulse with a TE/TR = 11.15/5000 ms, a saturation time of 3 s, a rare factor of 23 with a single average and 42 repetitions, a B_1_ of 1 μT, and an offset frequency step size of 0.4 ppm. Custom programs written in MatLab were used to generate MR images. For creating CEST maps, the magnetization transfer asymmetry (MTR_asym_) signal (Δ*S*/*S*_0_ defined as (*S*_-ω_ − *S*_+ω_)/*S*_0_) at frequencies ranging from 0.4 to 6.0 ppm (± 0.2 ppm) from water or the average signal of the entire frequency range (‘0.4–6.0’) was used. B_0_ inhomogeneity was corrected using the WASSR method as described before [31]. Regions of interest in phantoms, LNs, and lesions were manually selected based on T2-weighted images, where hyperintensities were not observed in naïve mice.

### MALDI imaging

Mice were euthanized with 5% isoflurane gas inhalation followed by thoracotomy, and the superficial cervical LNs were isolated and flash frozen (− 80 °C) until use. For MALDI mass spectrometry imaging, LNs were embedded in M1 media (Thermo Fisher), cryo-sectioned at 10 µm slice thickness and placed on indium tin oxide-coated glass slides (Delta Technologies). Tissue sections were warmed to room temperature (RT) in a vacuum desiccator for 10 min prior to spraying. 1,5-diaminonaphthalene (10 mg/ml) in 70% acetonitrile with 0.1% trifluoroacetic acid was applied using an HTX M5 sprayer (HTX Technologies) with the following parameters: 30-degree nozzle temperature, 4 passes, 0.1 ml/min flow rate, 1200 mm/min velocity, 2.5 mm track spacing criss-cross spray pattern, 10 psi pressure, and 2 l/min gas flow rate. MALDI imaging was performed in reflectron-negative mode at 100-micron pixel and raster size with 200 laser shots per pixel using a Bruker RapifleX MALDI TOF/TOF instrument. Candidate metabolites were identified by on-tissue MS/MS compared to MS/MS of pure standard compounds of metabolites on the RapifleX (Additional file 2: Data S1) and confirmed by high mass resolution imaging with mass accuracy < 1 ppm using a Bruker ScimaX 7T Fourier-transform ion cyclotron resonance instrument at the Bruker Daltonics Applications Lab (Billerica, MA, USA) (Additional file 3: Data S2). Adjacent sections were stained with H&E for histological referencing.

### Antigen-induced proliferation assay

Mice were euthanized with an overdose of 5% isoflurane followed by thoracotomy. Half of the spleen was strained using a 100 μm filter. Red blood cells in the single cell suspensions were lysed using 155 mM NH_4_Cl, 10 mM KHCO, and 0.1 mM EDTA). Single cell suspensions were cultured in 96-well plates (2 × 10^5^ cells/well) for 3 days with basal media supplemented with or without MOG_35-55_ (1 or 10 ng/ml). Basal media contained Dulbecco’s modified eagle medium/F12 (Gibco, 11330-032), fetal bovine serum (FBS, 10%; Hyclone, SH30070.03), penicillin–streptomycin (1%; Gibco, 15140-122), and beta-mercaptoethanol (1%; Gibco, 21985023). Proliferation index was defined as the cell number after 3 day culture in the presence of antigen divided by the cell number at 3 days without antigen.

### Flow cytometry

Mice were euthanized with an overdose of 5% isoflurane followed by cardiac perfusion with cold Hank’s balanced salt solution without Mg^2+^ and Ca^2+^ (HBSS). CNS tissue was mechanically dissociated with a 16G needle, then digested with collagenase IV (2 mg/ml) and DNAse (100 U/ml) in HBSS on a shaker rotator for 30 min at 37 °C with trituration halfway through digestion and at the end. Cells were then pelleted and resuspended in 30% Percoll and centrifuged at 550×*g* for 10 min. Myelin debris was aspirated off and cells were washed in HBSS. Superficial cervical LNs were mechanically dissociated and strained using a 100 μm filter. After preparation of single cell suspensions, cells were divided in half. Half of each sample was cultured in complete Iscove’s modified Dulbecco’s medium with a cell stimulation cocktail containing protein transport inhibitors (eBioscience, 00-4975-93). The other half was processed immediately for flow cytometry. Single cell suspensions were washed in 10 mM PBS pH = 7.3, then stained for live/dead cells (Miltenyi, 130–109-814) for 30 min at RT and antibody Fc block (Biolegend, 156604), for 10 min at 4 °C. Cells were stained for surface markers using fluorescent antibodies (Additional file 1: Table S1) for 30 min at RT in fluorescence activated cell sorting (FACS) buffer containing 2% FBS, 2 mM ethylenediaminetetraacetic acid in 10 mM PBS, pH = 7.3. For intracellular staining, cells were fixed with intracellular fixation buffer (eBioscience, 00-8222049) for 30 min, then washed in permeabilization buffer (PB, eBioscience, 00-8333-56), and stained with antibodies for 1 h at RT in the dark in PB. Cells were washed again with PB and with FACS buffer, followed by resuspension in FACS buffer for flow cytometry. Cellular staining was quantified using a MACSQuant 10 flow cytometer (Miltenyi Biotech) and data were analyzed using FlowJo software with standard gating schemes (Additional file 1: Figs. S1–S4).

### Statistical analysis

GraphPad software was used for all data analysis. A Spearman’s rank test was used to assess correlations. ANOVA with a Bonferroni post hoc test was used to evaluate comparisons. In all cases, *p* < 0.05 was considered statistically significant.

## Results

**Fig. 1:**
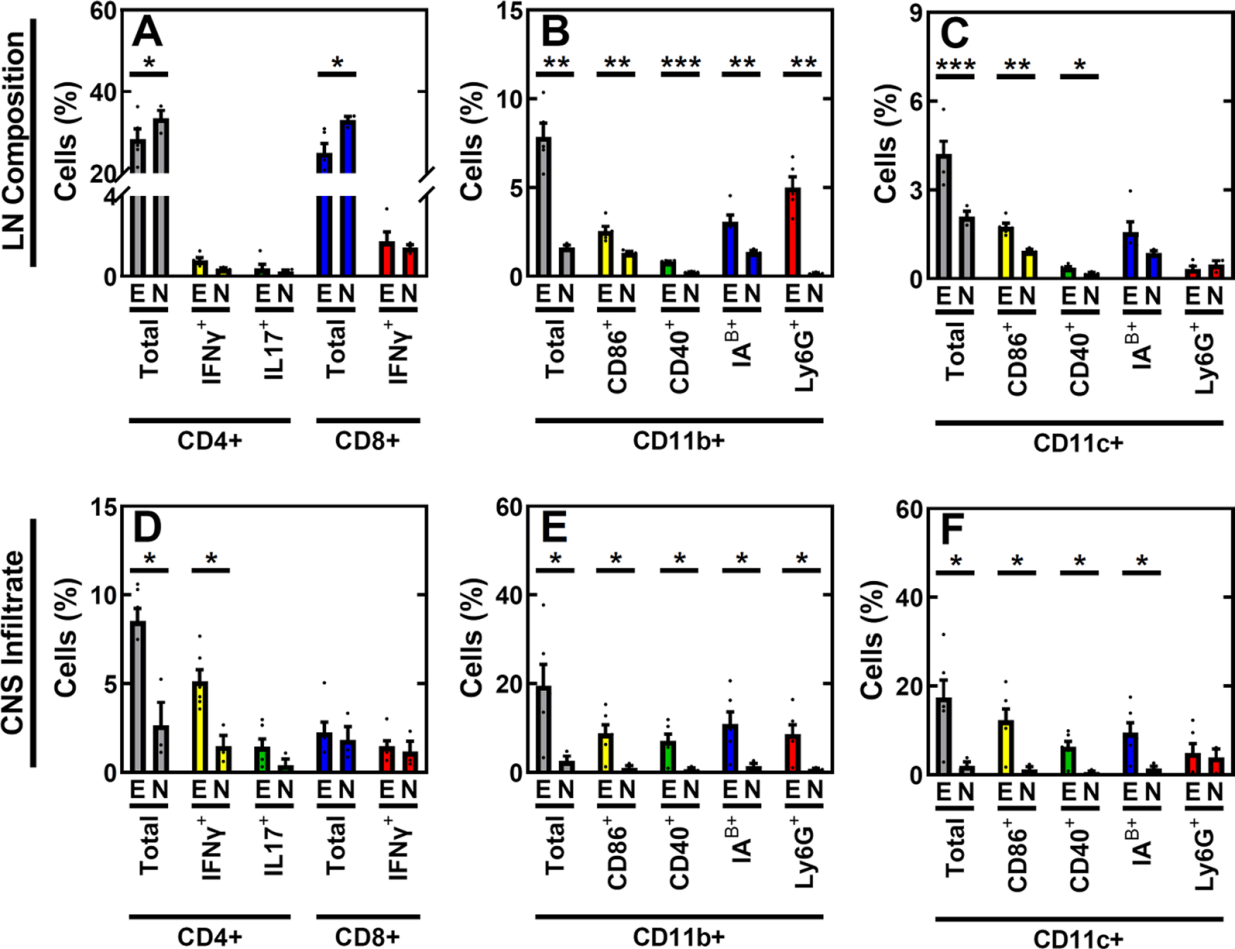
Altered immune cell profiles in EAE-induced mice at 14 DPI. Comparison of immune cell composition in (**A**–**C**) CNS-draining, superficial cervical LNs and (**D**–**F**) in CNS tissue of EAE (*n* = 5) and naïve (*n* = 3) mice. Presence (%) of established activation markers were assessed for (**A, D**) T cells gated using CD3 + , and (**B, E**) myeloid cells and (**C, F**) dendritic cells gated using CD45 + in LNs and CNS tissue. E = EAE mice, N = naïve mice. *p < 0.05, **p < 0.01, ***p < 0.001 using ANOVA with Bonferroni post-hoc

We first evaluated alterations in the cellular composition of the CNS and superficial cervical LNs in EAE mice using flow cytometry (Fig. 1). Antigen-presenting cells (APCs) were defined as CD45 + /CD11b + cells in LNs and CD45^HI^/CD11b + in the CNS. T cells were defined as CD3 + /CD4 + and CD3 + /CD8 + cells for both tissues. Markers evaluated to identify mature CD11b + APCs included CD40+, CD86+, Ly6g + and IA^B^ + . Markers evaluated to identify effector T cells included IFNγ + , CD44 + /CD62L− and IL17a + (for CD3 + /CD4 +). The presence of lymphocytes and activation markers significantly differed between EAE and naïve mice, similar to previously reported trends for this model [34]. In the superficial cervical lymph nodes, the presence of CD11b + and CD11c + APCs in EAE mice were 387% and 101% higher than that of naïve mice, while the presence of CD4 + and CD8 + T cells were 15% and 24% lower in EAE mice, respectively. The presence of CD11b + and CD11c + APCs with mature markers CD86 and CD40 were significantly (*p* < 0.05) higher in EAE mice, while the presence of IFNγ + and IL17a + T cells were similar between the two groups. Ly6g + and IA^B^ + CD11b + cells, but not Ly6g + and IA^B^ + CD11c + cells, were also higher in the EAE group. EAE mice also had a 659% and 762% higher CNS infiltration by CD11b + and CD11c + APCs and 223% higher CNS infiltration by CD4 + T cells, respectively, of CNS tissue (*p* < 0.05). CD8 + T cell infiltration of CNS tissue was also higher in EAE mice (24%), but not significant (*p* > 0.05).

We then evaluated CEST signal of phantoms that contained biomolecules associated with activated immune cells and inflammation at multiple frequencies ranging from 0.4 to 6.0 ppm (± 0.2 ppm), based on their sensitivities to molecules: lactate, glucose, glutamate, and proteins, such as albumin (Additional file 1: Fig. S5). We conducted our investigations with a saturation field strength (B_1_) of 1 μT because of its demonstrated use in detecting protons in these molecules (Additional file 1: Fig. S5) at this field strength of 11.7T, despite the presence of appreciable relayed NOEs and some MTC effects at the low-frequency part of the Z-spectrum [35].

**Fig. 2:**
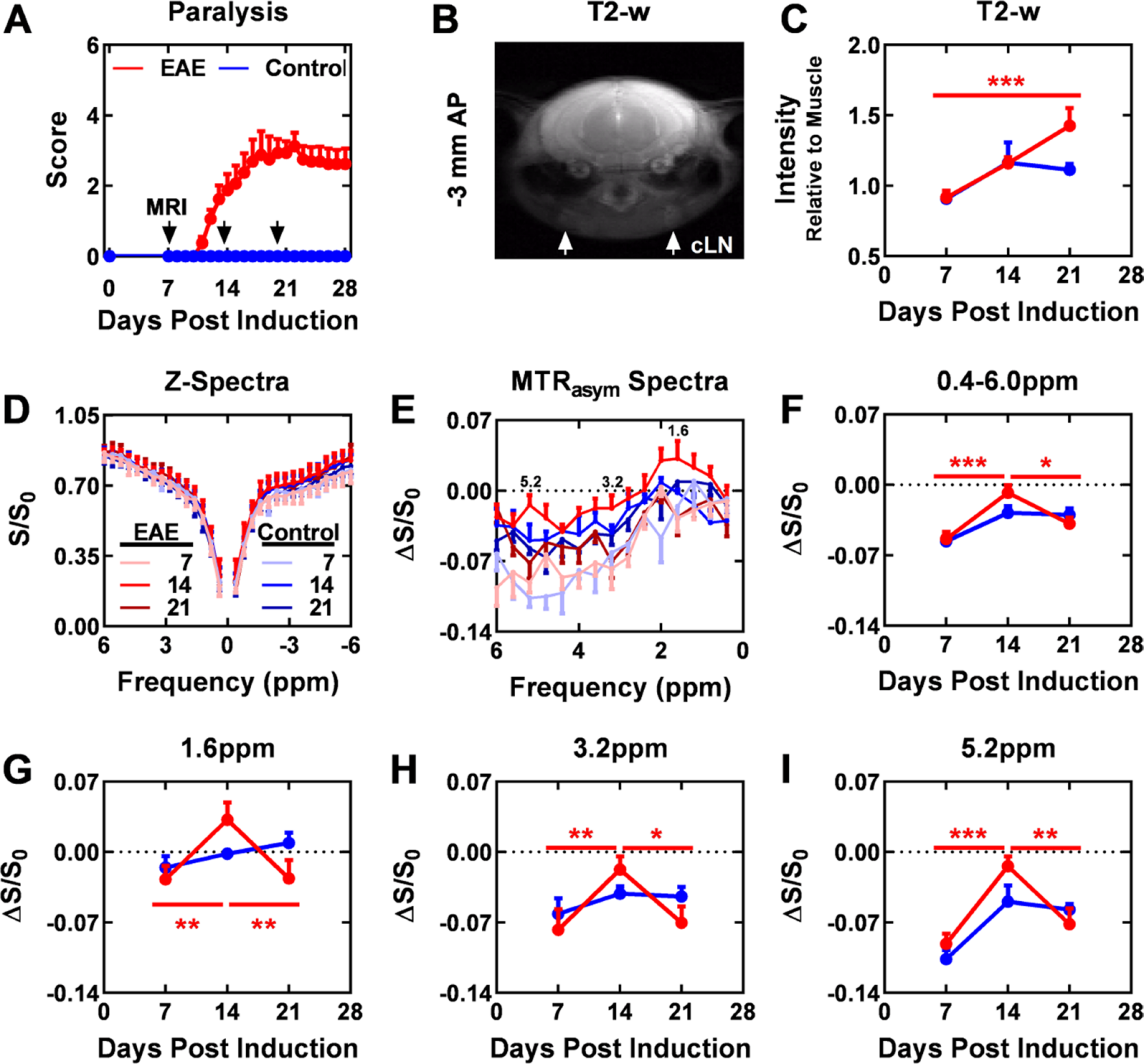
MRI of CNS-draining, superficial cervical LNs during EAE progression. **A** Disability score during the course of EAE. Arrows highlight days MRI was performed. **B** Visualization of the LNs using T2-weighted (T2-w) MRI. Quantification of (**C**) T2-w MRI and (**D, E**) CEST MRI signal intensity as disability progressed. Frequency ranges (± 0.2 ppm) whose average MTR_asym_-based CEST signals in EAE-induced mice (*n* = 8) and not in control mice (*n* = 4) significantly (*p* < 0.05) differed include (**F**) 0.4–6.0, (**G**) 1.6, (**H**) 3.2, and (**I**) 5.2 ppm. *cLN* cervical LN. *p < 0.05, **p < 0.01, ***p < 0.001 using ANOVA with Bonferroni post-hoc

**Fig. 3:**
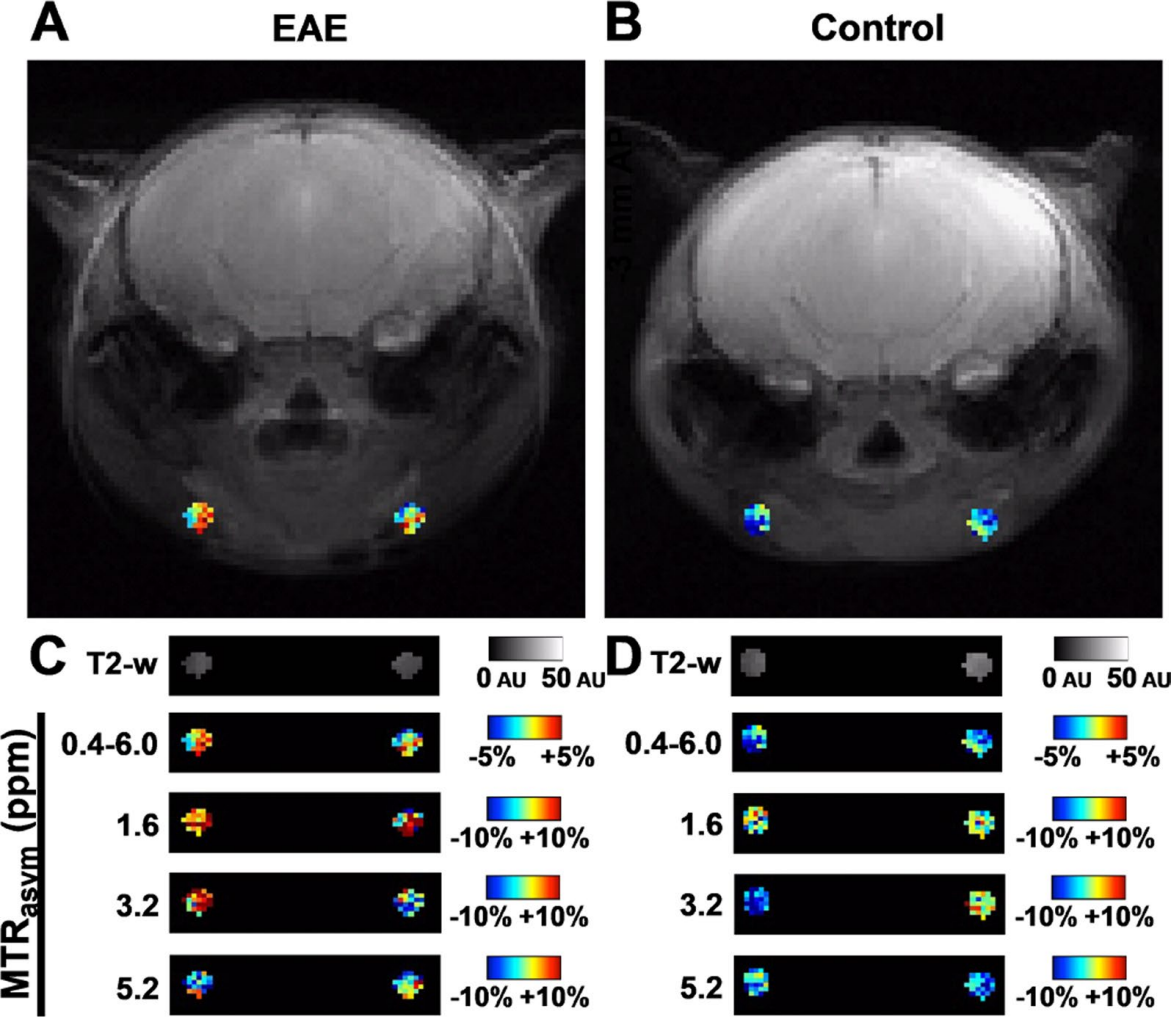
MRI of CNS-draining, superficial cervical LNs at 14 DPI. Visualization of MR signal in (**A, C**) EAE-induced and (**B, D**) control (no MOG_35-55_) mice. **A, B** Overlay of average MTR_asym_-based CEST map (0.4–6.0 ppm) of LNs on T2-w image for anatomical reference. **C, D** Isolated T2-w and average MTR_asym_-based CEST maps at frequency ranges (± 0.2 ppm) with significant (*p* < 0.05 using ANOVA with Bonferroni post-hoc) alterations of signal in EAE-induced mice and not in control mice: 0.4–6.0, 1.6, 3.2, and 5.2 ppm

We next investigated the ability of CEST MRI of superficial cervical LNs to predict and monitor the initiation and resolution of a paralyzing inflammatory attack in the EAE model. Mice were imaged when the majority of mice were before disease onset (7 DPI, paralysis score ≤ 1), as paralysis was progressing (14 DPI), and after paralysis stabilized (21 DPI) (Fig. 2a). We imaged the coronal slice of the cerebral brain approximately 3.5 mm posterior from bregma to permit monitoring superficial cervical LNs (Fig. 2b). MRI metrics in superficial cervical LNs were dynamic in EAE mice (Fig. 2c–i). Prior to paralysis onset (7 day DPI), T2-w lesions were not visible in these mice and CEST signals in superficial cervical LNs were similar to naïve controls. At the acute stage (14 DPI), onset of paralysis (clinical score ≥ 1) had occurred for 6 out of 8 mice, of which 4 had visible lesions on T2-w MR images and 3 had peaked on the day of imaging. At this time, MTR CEST signal (Δ*S*/*S*_0_) in LNs of EAE-induced mice at the 1.6, 3.2, and 5.2 ppm frequencies significantly increased from 7 DPI in EAE mice, but not in control mice. Alterations in CEST signal (*S*/*S*_0_) were not observed at any frequency in the Z-spectra (− 6 to + 6 ppm). The T2-w signal in both EAE and control mice had increased from 7 DPI, albeit not significantly, which may reflect an artifact of the inflammation that occurs as part of the induction process of the model. MTR_asym_ signal alterations in these LNs were not symmetric at 14 DPI (Fig. 3). By the chronic stage (21 DPI), the remaining (2 out of 8) EAE mice that were not paralyzed 14 DPI were now experiencing paralysis and 1 had visible white matter lesions. MTR_asym_ signal at the 1.6, 3.2, and 5.2 ppm frequencies was significantly lowered from 14 DPI. This decrease in MTR_asym_ signal is consistent with reports that the activation state of immune cells in the LNs of EAE mice at the chronic stage is lower compared to those in the active stage [36]. At this time, all CEST MRI metrics in the LNs of EAE mice were similar to control mice; however, T2-w signal in the LNs at 21 DPI was significantly higher than at 7 DPI in EAE mice, but not in control mice. We continued to investigate the origins of the CEST signals at 1.6, 3.2, and 5.2 ppm based on their significant, and transient, alterations in the LN of EAE mice and not control mice. At 14 DPI, the two indicators of disease activity, the presence of T2-w lesions and increasing paralysis, was observed for the majority of mice (6 out of 8).

**Fig. 4:**
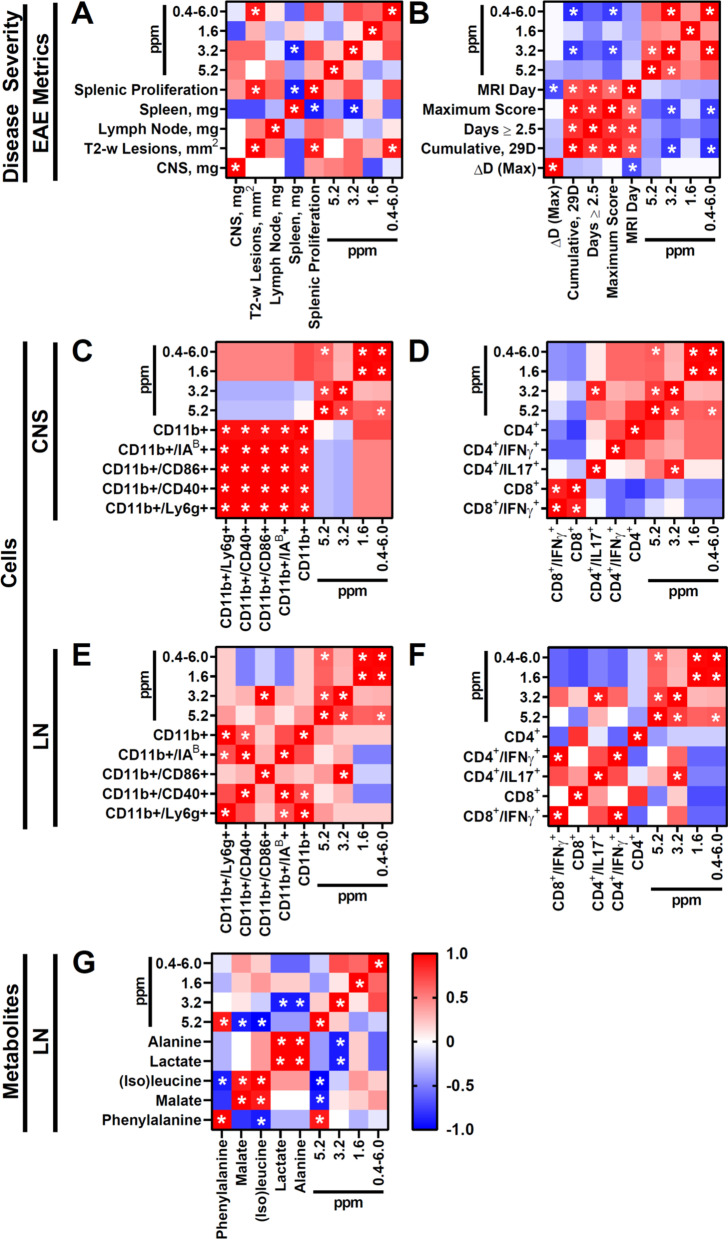
Spearman's rank correlation of MTR_asym_ signal in EAE-induced mice (13–14 DPI) to immune cell composition (14–15 DPI). Comparison to (**A**) immune cell activity (14–15 DPI; *n* = 5) and to (**B**) paralysis severity (29 DPI; *n* = 8). Comparison to (**C**–**F**) immune cell (T cell, myeloid cell; 15 DPI) profile (%) in (**C, D**) CNS tissue (*n* = 6) and in (**E, F**) CNS-draining, superficial cervical LNs (*n* = 5). **G** Comparison to metabolites in CNS-draining, superficial cervical LNs (14–15 DPI; *n* = 5). *p < 0.05

We compared CEST imaging to more established metrics for monitoring disease progression and immunological activity in EAE and MS (*n* = 5–8) (Fig. 4a, b). We chose to analyze the acute phase of the EAE model (13–14 DPI), as hyperintense T2-w lesions can be observed and paralysis is still progressing. CNS damage metrics included CNS size (atrophy) and T2-w hyperintense lesions in the brain (*n* = 5). These metrics did not correlate with each other (*ρ* = 0; *p* > 0.05) (Fig. 4a). Disability metrics included current, cumulative, and maximum paralysis score; number of severe (score ≥ 2.5) days; and number of days before maximum score (*n* = 8) (Fig. 4b). The disability severity metrics (scores, number of severe days) metrics largely correlated with each other (+ 0.56 ≤ *ρ* ≤  + 0.92; 0.002 < *p* < 0.48). Immunological activity metrics included secondary lymphoid organ size (spleen, LNs) and antigen-induced immune stimulation of splenic cells (proliferation index). Interestingly, CEST signal at 3.2 ppm and proliferation index correlated with spleen size (ρ = − 9.00; *p* = 0.04 for both), but not LN size (− 0.10 ≤ *ρ* ≤  + 0.60; *p* > 0.05).

We next compared LN CEST imaging to immune cell presence and phenotype in the CNS (*n* = 6) and in superficial cervical LNs (*n* = 5) (Fig. 4c–f). CEST did not correlate with total APC presence (0.60 ≤ *ρ* ≤  + 0.71; *p* > 0.05) (Fig. 4c) or T cell presence (+ 0.03 ≤ *ρ* ≤  + 0.33; *p* > 0.05) (Fig. 4d) in the CNS either. LN CEST signal did not correlate with total APC presence (-0.50 ≤ *ρ* ≤  + 0.40; *p* > 0.05) (Fig. 4e) or T cell presence (-0.70 ≤ *ρ* ≤  + 0.20; *p* > 0.05) (Fig. 4f) at any of the evaluated frequencies. The LN CEST signal at 3.2 ppm correlated with mature APC presence (CD11b + /CD86 + ; *ρ* =  + 1.00; *p* = 0.008) and effector T cell presence (CD4 + /IL17a + ; *ρ* =  + 0.90; *p* = 0.04) in the LN. LN CEST signal at the 3.2 ppm frequency did not correlate with mature APC presence (CD11b + /CD86 +  = − 0.31 ≤ *ρ* ≤  + 0.71; *p* > 0.5) but did correlate with effector T cell presence (CD4 + /IL17a + ; *ρ* =  + 0.89; *p* = 0.02) in the CNS. Surprisingly, mature markers for APCs did not necessarily correlate with each other in the LNs (− 1.00 < *ρ* <  + 1.00; 0.01 < *p* < 0.39), but largely correlated in the CNS (+ 0.71 < *ρ* <  + 1.00; 0.001 < *p* < 0.7). Effector markers for T cells also did not reliably correlate with each other in LNs (− 0.70 ≤ *ρ* ≤  + 1.00; 0.008 < *p* < 0.53) or in the CNS (− 0.77 ≤ *ρ* ≤  + 1.00; 0.001 < *p* < 0.50). These phenomena may reflect differences in the regulation of these markers.

**Fig. 5:**
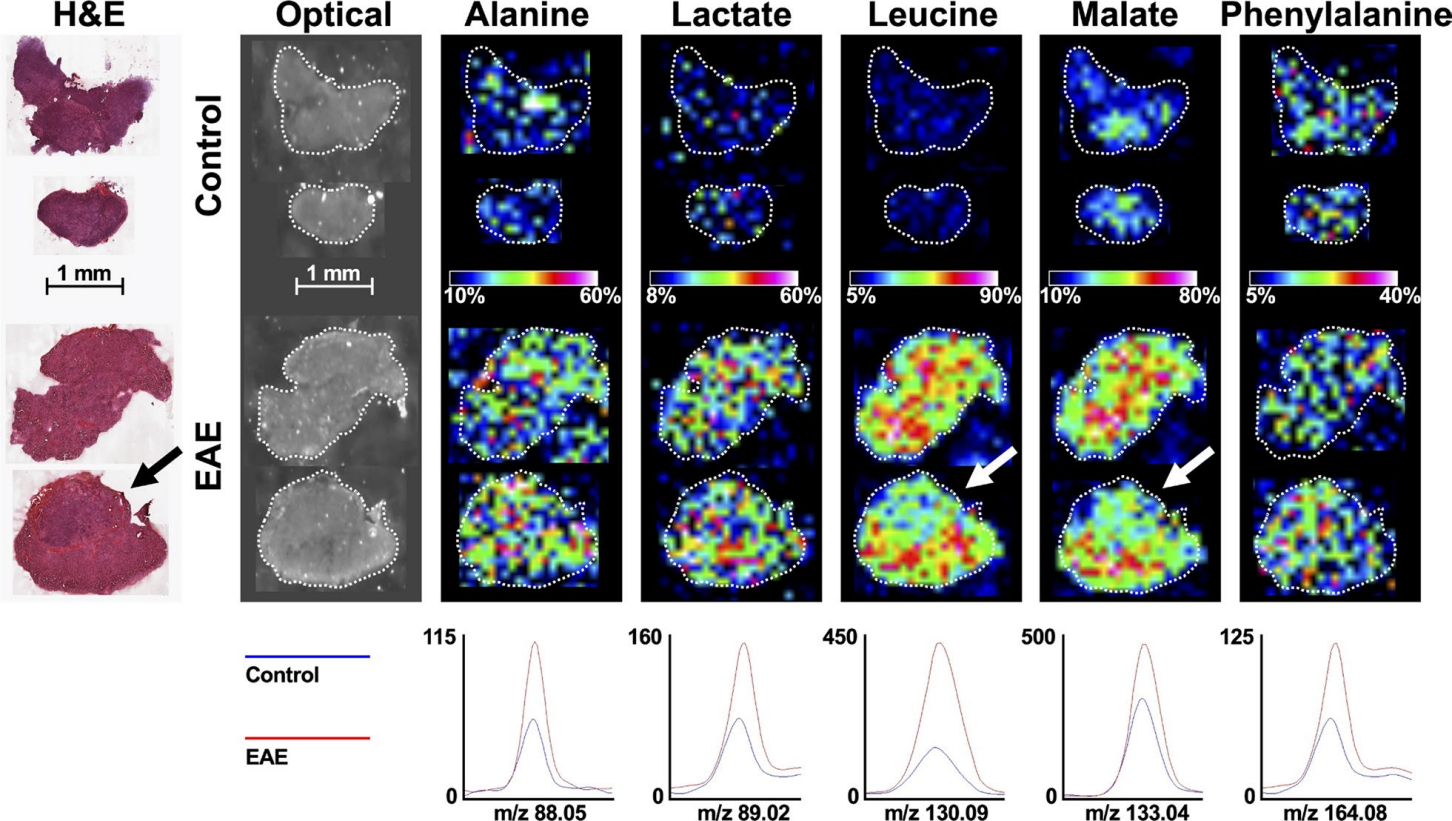
MALDI imaging of the top 5 most significantly altered metabolites that correlate with MTR_asym_ signal in EAE-induced mice at 13–14 DPI. Visualization of alanine, lactate, leucine, malate and phenylalanine presence in representative cLNs of (top row) control and (middle row) EAE mice. (Bottom row) Average (*n* = 5) signal intensity of these metabolites at their characteristic mass-to-charge ratio (*m*/*z*). Optical imaging is shown to highlight the location of the cLNs. H&E staining is shown to highlight intra-nodal regions (medulla and cortex). Arrows highlight prominent examples of these regional differences

We then compared CEST imaging of these LNs to their molecular composition using MALDI mass spectrometry imaging (Fig. 4g). We screened 13 metabolites that have intracellular concentrations [37–39] and water-exchanging moieties [40–44] with potential for altering CEST MRI signal: glycine, alanine, lactate, serine, proline, threonine, (iso)leucine, aspartate, malate, glutamine, glutamate, phenylalanine, and glucose (ordered by mass). Of these metabolites, glycine, alanine and lactate negatively correlated with the 3.2 ppm CEST signal (*ρ* = − 0.90; *p* = 0.04). Phenylalanine positively correlated with the 5.2 ppm CEST signal (*ρ* = 0.90; *p* = 0.04), while (iso)leucine and malate negatively correlated with the 5.2 ppm CEST signal (− 1.00 ≤ *ρ* ≤ − 0.900; 0.008 < *p* < 0.04). None of these metabolites correlated significantly with the 1.6 ppm CEST signal. The average levels of the top 5 metabolites alanine, lactate, (iso)leucine, malate and phenylalanine were elevated in LNs of EAE mice as compared to control mice, though not significantly (*p* > 0.05) (Fig. 5).

## Discussion

The primary metrics for evaluating disease progression and therapeutic efficacy in MS are antigen-induced immune cell stimulation, MRI-mediated visualization of CNS damage, and disability assessment. When the antigen is known, the former metric corresponds well temporally with immunological attack and also correlates strongly with future disability [45]. However, these antigens vary greatly amongst patients, expand in number over time, and are difficult to identify [46], reducing their practicality. To circumvent the need to identify the auto-antigens, MRI is performed to monitor CNS damage, e.g., lesions and atrophy. Visualization of lesions using MRI has been a principle component of diagnosis and monitoring of MS for decades [47]. Three scans are routinely performed on MS patients to detect lesions: gadolinium (Gd)-enhanced, T1-w, and T2-w MRI. Gd-enhanced MRI, which visualizes disruption of the BBB, can detect de novo lesions [48], although recent reports have raised pharmacokinetic concerns regarding its repeated use [49, 50]. T1-w and T2-w MRI, which are sensitive to brain water and fat content, can visualize lesions not enhanced by Gd-enhanced conventional MRI.

Meta-analyses have revealed that monitoring CNS damage using MRI is a poor predictor of disease progression in MS, likely because variation in the resulting damage (shape, location, size) obscures its consequence [51, 52]. Clinically, MRI quantification of lesion load weakly correlates with immune responses (0.1 < *ρ* < 0.6) [53, 54] and future disability (0.1 < *ρ* < 0.5) [55, 56]. In the EAE model, we observed that lesion load in the brain (mm^2^) predicted immune response (*ρ* =  + 0.90; *p* < 0.05), but not disability (+ 0.36 ≤ *ρ* ≤  + 0.87; *p* > 0.05). Recent evidence emerging from quantitative MRI and post-mortem pathology has revealed that MS is a neurodegenerative disorder primarily consisting of widespread, diffuse damage with sporadic sclerotic lesions [57–59], resulting in cell loss [60] and atrophy [61]. Molecular imaging studies in the EAE model further support the existence of diffuse damage [18, 61, 62], yet similarly to focal lesions, the shape and location of diffusely damaged tissue during immunological attack is unpredictable [62]. We observed correlation between CNS size and paralysis to be similarly low (− 0.15 ≤ *ρ* ≤ 0.45; *p* > 0.05) to that in MS (− 0.4 < *ρ* <  + 0.4) [63, 64].

To identify which cells may be responsible for the CEST signal changes, we performed flow cytometry, which enabled us to examine 15 types of immune cells that play a role in MS and its preclinical models. CEST signal at the 1.6 and 5.2 ppm frequency did not correlate with any of these immune cell subpopulations. However, CEST signal at the 3.2 ppm frequency correlated with LN composition (CD11b + /CD86 + and CD4 + /IL17a + cells) and CNS infiltration of immune cells (CD4 + /IL17a + cells). CD11b + APCs have been demonstrated in multiple models to be initiators and potent drivers of MS [65, 66]. CD11b + APCs consist of CD11c-/CD11c^LO^ macrophages/monocytes and CD11c + dendritic cells. More CD11b + APCs and CD11c + dendritic cells were present in the LN and CNS of EAE mice than the control group. Furthermore, the costimulatory molecule CD86 is a well-established marker of activation for APCs and dendritic cells [67, 68] whose expression increases with progression of disease in EAE [69]. More recently, CD4 + /IL17a + T-cells have been shown to be important primers of EAE, enhancing the severity of disease [70]. Antigen-specific expression of IL-17 has been documented in the LN and the CNS for this EAE model [71]. Furthermore, the presence of CD4 + /IL17a + T-cells in the CNS has been shown to play a role in the recruitment of activated CD11b + cells to the CNS at early stages of inflammatory attack [72].

Earlier (prodromal) stages of MS pathophysiology induce metabolic changes, e.g., glutamate [73, 74] and lactate [7, 75, 76], which are metabolites that can be detected using CEST MRI [41, 77]. Previously we utilized CEST MRI to monitor diffuse changes in the brain and spinal cord that preceded [78] or developed in absence [31] of lesion formation. In this report, we utilized CEST MRI to evaluate the molecular composition (signal at a specified frequency range, ΔS/S_0_) of LNs as a potential imaging biomarker for MS progression. We demonstrated that changes in CEST signal in CNS-draining, superficial cervical LNs (0.4–6.0, 1.6, 3.2, and 5.2 ppm) corresponded with disability progression. Interestingly, we observed that the 3.2 ppm CEST frequency was sensitive to amine-containing compounds, particularly those in amino acids and proteins, as demonstrated using phantom imaging (Additional file 1: Fig. S5). Activated immune cells alter their amino acid metabolism to accommodate higher energy demands [79]. Macrophages exposed to lipopolysaccharide (LPS) have higher intracellular glutamate [80, 81]. Dendritic cells also increase their secretion of glutamate [3] when exposed to LPS. Altered glutamate regulation has been observed in both MS and EAE. Glutaminase, the enzyme responsible for converting glutamine to glutamate, is expressed at higher levels in MS lesions [82] and in the spinal cords of EAE animals [83], and is attributed to alterations in macrophage and T cell metabolism [9, 84]. Amino acids induce a strong CEST MRI signal, but some have overlapping saturation frequencies making the CEST signal not metabolite-specific but metabolite-sensitized or -weighted.

To investigate which specific metabolites may be responsible for the CEST signal changes, we applied MALDI imaging, a technique that can identify specific molecules and metabolites based on a laser energy absorbing matrix that creates ions from large molecules with minimal fragmentation. MALDI imaging mass spectrometry has entered the field of tissue-based research by providing unique advantages for analyzing tissue specimen in unprecedented detail. This method is label-free and allows multiplex analysis of hundreds to thousands of molecules in the very same tissue section simultaneously. A broad spectrum of analytes—many of which are potential CEST MRI targets—ranging from proteins, peptides, protein modification over small molecules, drugs and their metabolites as well as pharmaceutical components, endogenous cell metabolites, lipids, and other analytes are made accessible by this in situ technique [85]. We compared the metabolic information from MALDI tissue imaging with the CEST MRI signals. MALDI imaging revealed that most amino acids that could potentially contribute to CEST signal at the 3.2 ppm frequency (serine, proline, threonine, (iso)leucine, aspartate, glutamine, glutamate, phenylalanine) did not correspond with CEST signal at this frequency. Furthermore, glycine and alanine negatively correlated with CEST signal levels. None of these metabolites correlated with CEST signal at the 1.6 ppm frequency, while phenylalanine (positively), malate (negatively), and (iso)leucine (negatively) correlated with CEST signal at the 5.2 ppm frequency.

Using MALDI imaging we showed that CEST imaging trends were not entirely explained by changes in free metabolite content. We recognize that in addition to exchangeable proton concentration (i.e., biomolecular concentration), CEST is highly sensitive to T_1_ relaxation [86] and pH [87], both of which would need to be calculated to quantify the presence of these biomolecules using CEST MRI. In addition, the MTR_asym_-based CEST signal examined here has contributions from both metabolites (positive frequencies) and macromolecules that generate relayed NOEs (negative frequencies) and MT effects (an asymmetric effect impacting both positive and negative frequencies), which would also need to be accounted for. The negative values support the presence of a substantial NOE effect in the MTR_asym_-based CEST values observed, which may be mitigated through further optimization of saturation parameters, e.g., B_1_. We postulate, instead, that alterations in macromolecules that contain these metabolites, e.g., proteins, may be responsible in part for the changes in MTR_asym_-based CEST images due to EAE. Currently, there is limited information on which proteins and other macromolecules are present in concentrations high enough to individually contribute to CEST signal. However, elevated levels of glutamate-rich molecules have been observed in MS lesions using ^1^H MR spectroscopy [88, 89]. Furthermore, expression of ERICH-1, a glutamate-rich protein with a role in cell proliferation, has been reported to be enhanced in the T cells of MS patients [90]. Future studies utilizing “big data” technologies such as MALDI imaging along with artificial intelligence analysis may elucidate the molecular origins of the CEST MRI signal.

## Conclusions

We explored CEST imaging of CNS-draining, superficial cervical LNs, a key site for the activation and sensitization of immune cells responsible for MS, as a noninvasive strategy to monitor disease activity in a mouse EAE model with comparison to traditional biomarkers and metabolic changes identified using MALDI imaging. Previous imaging biomarkers developed by us and others have expanded the various forms of CNS damage in MS that can be noninvasively monitored, and the features of damaged regions that can be visualized. To the best of our knowledge, we are the first to report molecular imaging of secondary lymphoid organs as a strategy for monitoring an inflammatory disease. While the exchange regime for 11.7T was sufficiently slow to allow detection of these metabolites, saturation parameters will need to be further optimized to refine signal sensitivity at clinically relevant field strengths, wherein CEST MRI of MS-associated LNs may serve as a complementary imaging biomarker to monitor disease activity in MS.

## Supplementary Information

**Additional file 1:** **Figure S1**. Flow cytometry of APCs in CNS tissue. **Figure S2**. Flow cytometry of APCs in LN tissue. **Figure S3**. Flow cytometry of T cells in CNS tissue. **Figure S4**. Flow cytometry of T cells in LN tissue. **Figure S5**. MRI of metabolites altered in MS lesions and activated immune cells. Table S1. Antibodies used for flow cytometry.

**Additional file 2: **Standard target plate and on tissue MS-MS experiments performed at the Johns Hopkins Applied Imaging Mass Spectrometry (AIMS) Core.

**Additional file 3: **High mass resolution MALDI imaging experiments performed at the Bruker Daltonics applications laboratory in Billerica, MA.

## Data Availability

All data generated or analysed during this study are included in this published article and its Additional files, or in the Mendeley repository: https://doi.org/10.17632/ww4nnpm4kj.1.

## Abbreviations

APCs: Antigen-presenting cells
BBB: Blood–brain–barrier
CNS: Central nervous system
DPI: Days post-induction
EAE: Experimental autoimmune encephalomyelitis
FACS: Fluorescence activated cell sorting
HBSS: Hank’s balanced salt solution
LN: Lymph nodes
MALDI: Matrix-assisted laser desorption/ionization
MOG: Myelin oligodendrocyte glycopeptide
MRI: Magnetic resonance imaging
MRS: Magnetic resonance spectroscopy
MS: Multiple sclerosis
PB: Permeabilization buffer
