# Supplementary material for: CEST MRI and MALDI imaging reveal metabolic alterations in the cervical lymph nodes of EAE mice
Source: J Neuroinflammation. 2022 Jun 3;19:130. doi: 10.1186/s12974-022-02493-z (PMC9164344; doi:10.1186/s12974-022-02493-z)
Supplement: Supplementary file 1 — Additional file 1: Figure S1. Flow cytometry of APCs in CNS tissue. Figure S2. Flow cytometry of APCs in LN tissue. Figure S3. Flow cytometry of T cells in CNS tissue. Figure S4. Flow cytometry of T cells in LN tissue. Figure S5. MRI of metabolites altered in MS lesions and activated immune cells. Table S1. Antibodies used for flow cytometry. [file 12974_2022_2493_MOESM1_ESM.docx]

**Supplementary Information for**

**CEST MRI and MALDI Imaging Reveal Metabolic Alterations in the Cervical Lymph Nodes of EAE Mice**

Aline M. Thomas,^1,2^ Ethan Yang,^1^ Matthew D. Smith,^3,4^ Chengyan Chu,^1,2^ Peter A. Calabresi,^3,4^

Kristine Glunde,^1,5,6^ Peter C.M. van Zijl,^1,7^ Jeff W.M. Bulte,^1,2,6-9*^

**Author Affiliations**

^1^ Russell H. Morgan Department of Radiology and Radiological Science, Division of MR Research, Johns Hopkins University School of Medicine, Baltimore, MD.

^2^ Cellular Imaging Section and Vascular Biology Program, Institute for Cell Engineering, Johns Hopkins University School of Medicine, Baltimore, MD.

^3^ Department of Neurology, Johns Hopkins University School of Medicine, Baltimore, MD.

^4^ Solomon H Snyder Department of Neuroscience, Johns Hopkins University School of Medicine, Baltimore, MD.

^5^ Department of Oncology, Johns Hopkins University School of Medicine, Baltimore, MD.

^6^ Department of Biological Chemistry, Johns Hopkins University School of Medicine, Baltimore, MD.

^7^ F.M. Kirby Research Center for Functional Brain Imaging, Kennedy Krieger Institute, Baltimore, MD.

^8^ Department of Biomedical Engineering, Johns Hopkins University School of Medicine, Baltimore, MD.

^9^ Department of Chemical & Biomolecular Engineering, Johns Hopkins University School of Medicine, Baltimore, MD.

**Corresponding Author**

Jeff W.M. Bulte, Ph.D.

Department of Radiology

The Johns Hopkins University School of Medicine

Broadway Research Building Rm 659

733 N Broadway

Baltimore, MD 21205

Phone: 443-287-0996

Fax: 443-287-7945

Email: jwmbulte@mri.jhu.edu

**This PDF file includes:**

Figures S1 to S5

Table S1

Legends for Datasets S1 to S2


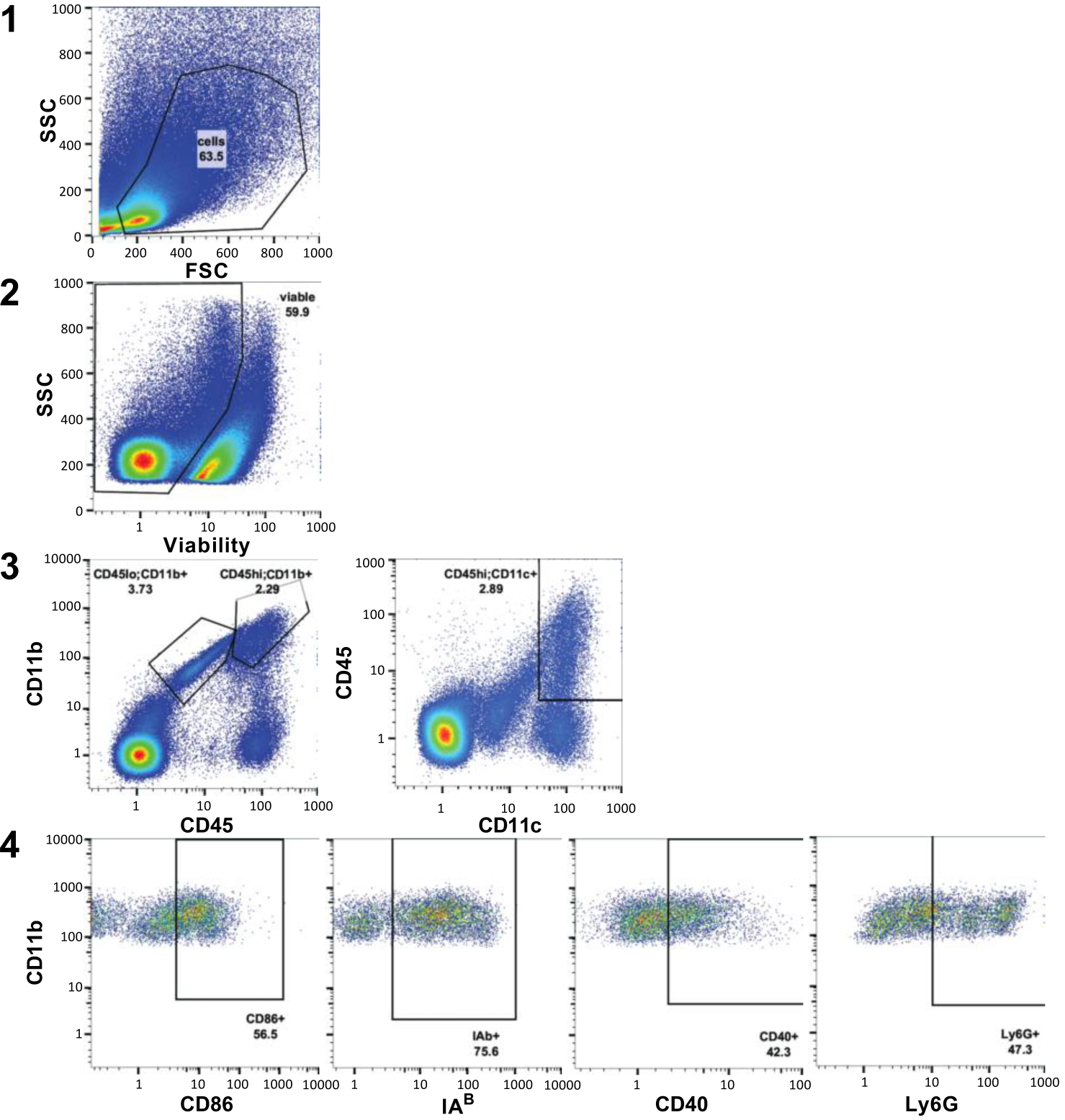


**Figure S1. Flow cytometry of APCs in CNS tissue.** Viable cells were identified using (**1**) side scatter (SSC) properties and (**2**) viability stain. (**3**) Discrimination of infiltrating CD45 high- (CD45^HI^) and resident low-expressing (CD45^LO^) APCs (CD11b+ cells). (**4**) APCs expressing immune activation markers.


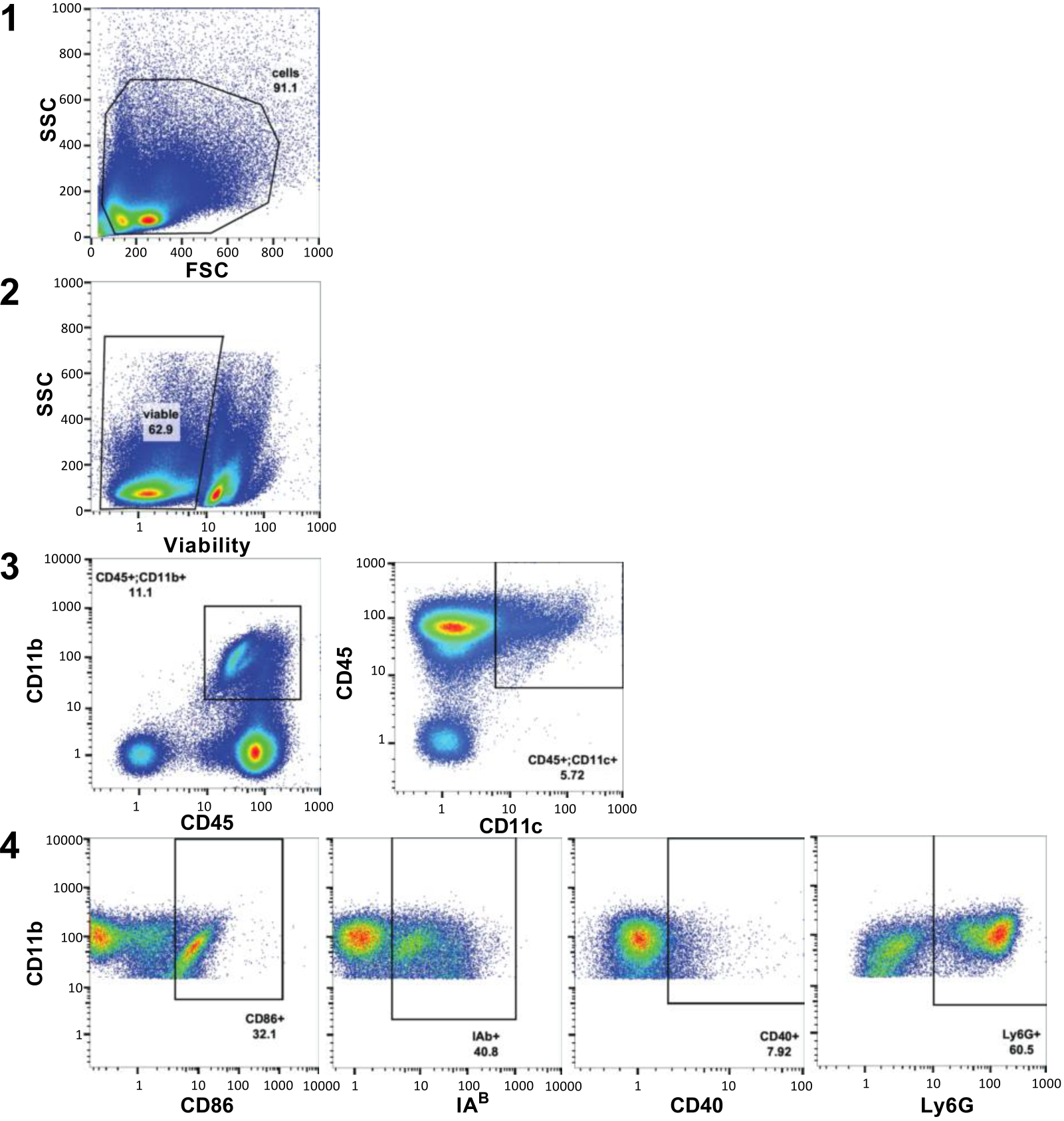


**Figure S2. Flow cytometry of APCs in lymphatic tissue.** Viable cells were identified using (**1**) side scatter (SSC) properties and (**2**) viability stain. (**3**) APCs expressing CD45 and CD11b. (**4**) APCs expressing immune activation markers.


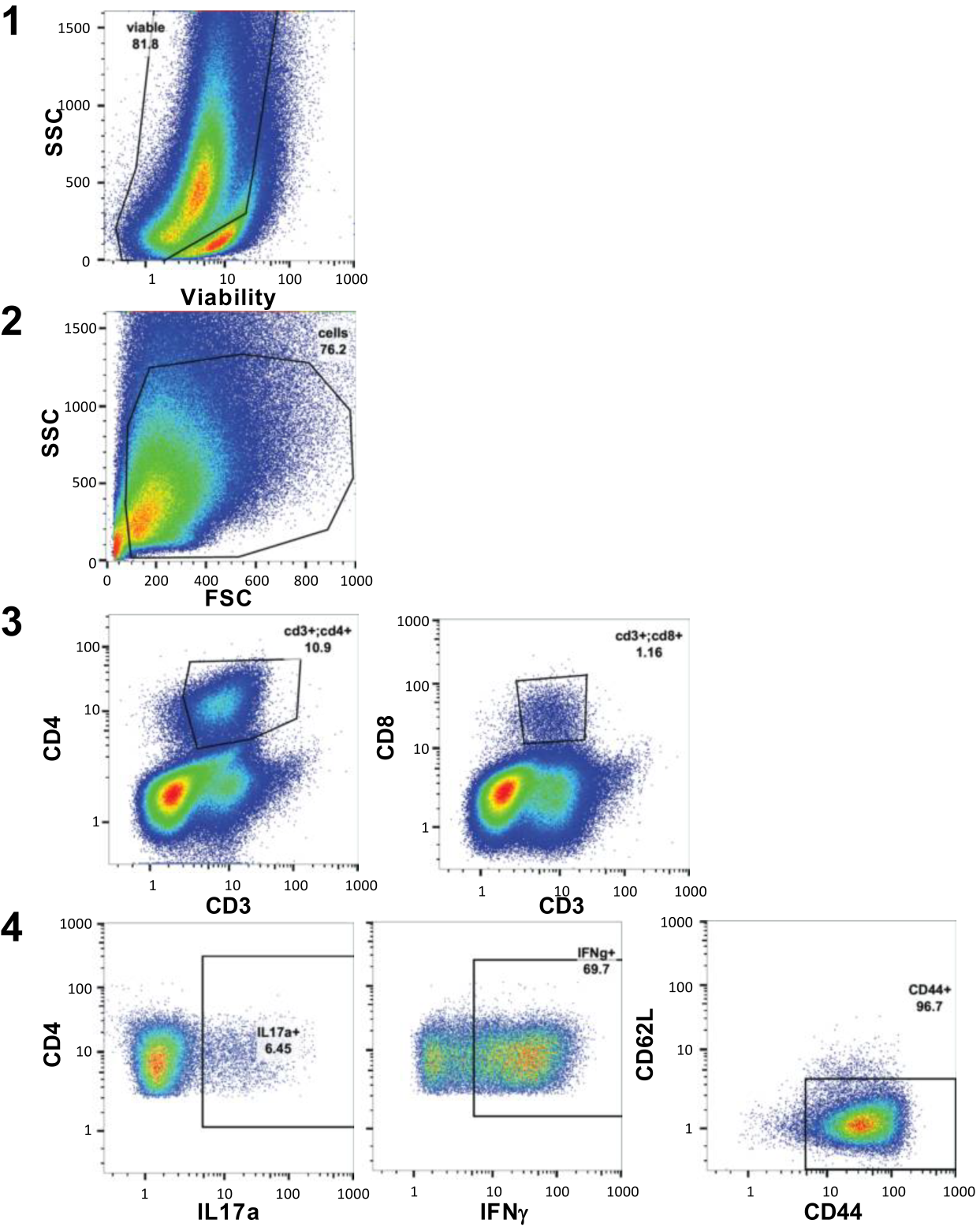


**Figure S3. Flow cytometry of T cells in CNS tissue.** Viable cells were identified using (**1**) side scatter (SSC) properties and (**2**) viability stain. (**3**) Discrimination of cytotoxic (CD4+) and helper (CD8+) T cells (CD3+). (**4**) T cells expressing immune activation markers.


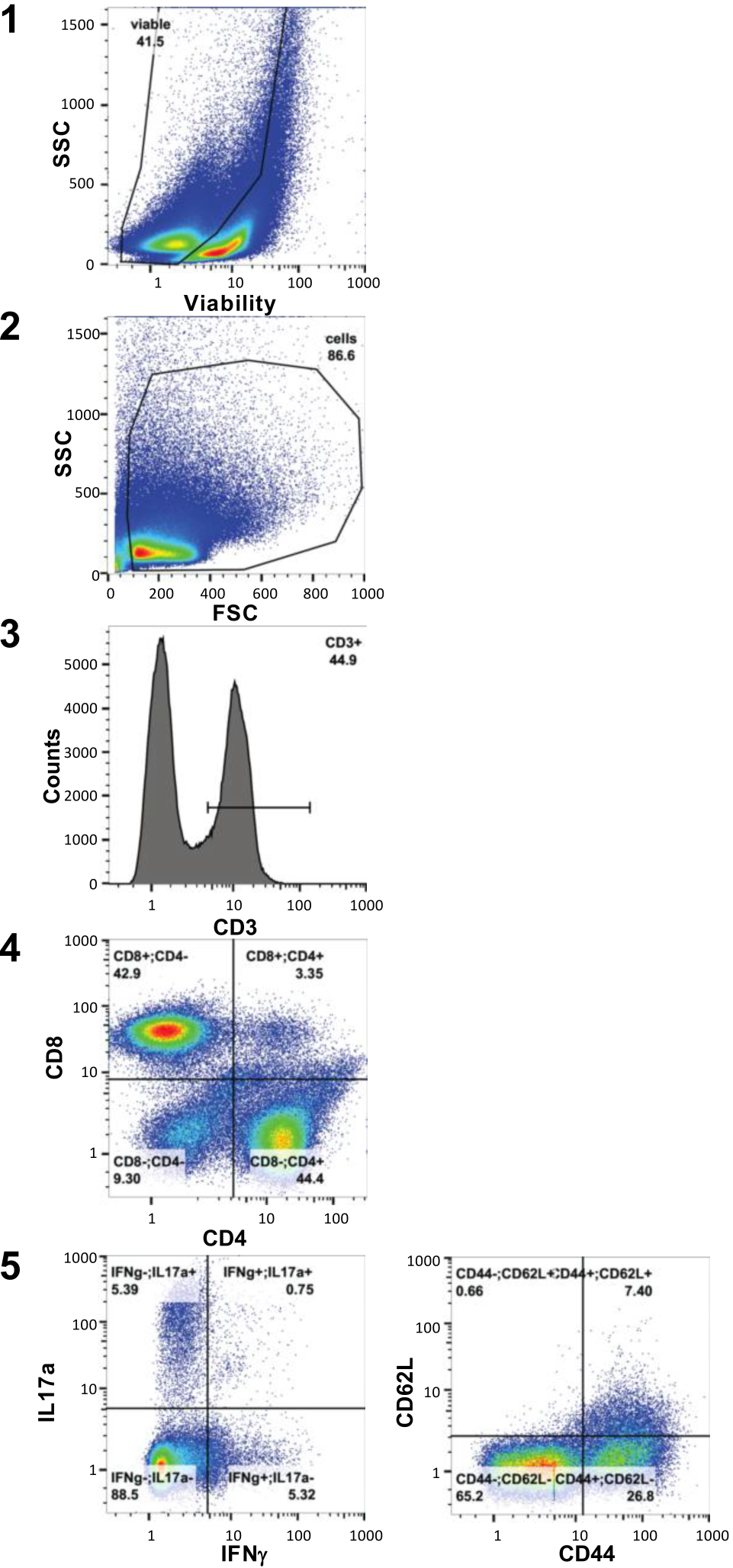


**Figure S4. Flow cytometry of T cells in lymphatic tissue.** Viable cells were identified using (**1**) side scatter (SSC) properties and (**2**) viability stain. (**3**) Representation of T cells (CD3+). (**4**) Discrimination of cytotoxic (CD4+) and helper (CD8+) T cells. (5) T cells expressing immune activation markers.


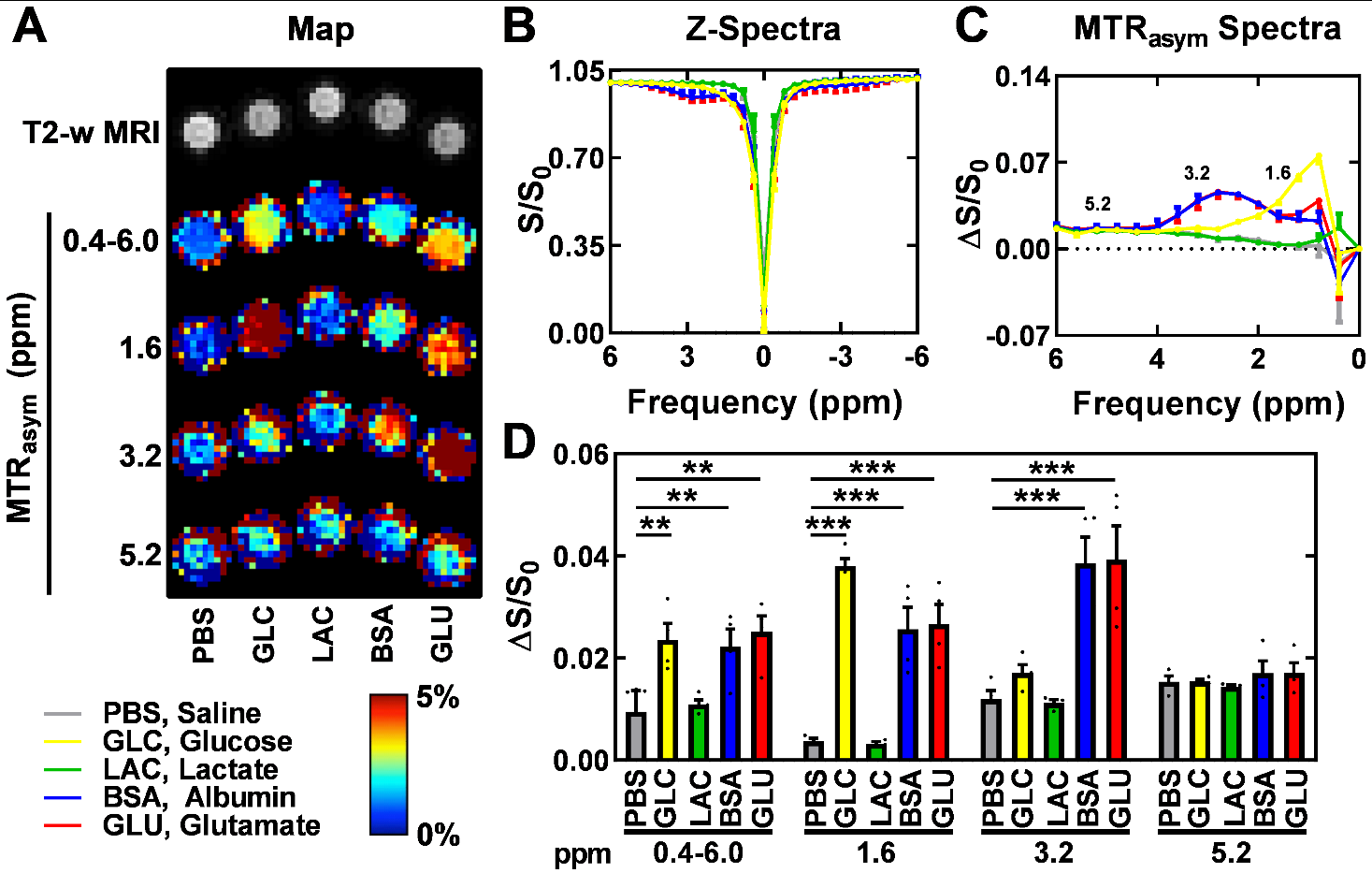


**Figure S5. MRI of metabolites altered in MS lesions and activated immune cells.** (**A**) Visualization and (**B-D**) quantification of (**B**) Z-spectral signal and (**A,C,D**) MTR_asym_ signals in agarose (0.25%) phantoms (*n*=3) containing glucose (50 mM), lactate (50 mM), albumin (5% w/v) or glutamate (50 mM) in PBS at pH = 7.3 and T = 25C (control). (**A,D**) Average MTR_asym_ signal at each saturation frequency or range of frequencies (0.4-6.0 ppm) with significant (*p*<0.05) alterations of signal in EAE-induced mice and not in control mice in *figure 2*. *=*p*<0.05, ** = *p*<0.01, ***=*p*< 0.001.

**Table S1. Antibodies used for flow cytometry**

| **Antigen** | **Clone** | **Conjugate** | **Vendor** | **Catalogue** |
| --- | --- | --- | --- | --- |
| TruStain Fc block | 93 | - | Biolegend | 156604 |
| Viability | - | 405/520 | Miltenyi | 130-109-814 |
| CD45 | 30-F11 | APCFire750 | Biolegend | 103154 |
| CD11b | M1/70 | eFluor450 | ThermoFisher | 48-0112-82 |
| CD11c | N418 | PECy7 | Biolegend | 117318 |
| CD86 | GL1 | PE | BD Biosciences | 553692 |
| CD40 | 1C10 | APC | eBioscience | 17-0401-82 |
| IA^B^ | M5/114.15.2 | PerCP | BioLegend | 107624 |
| Ly6G | RB6-8C5 | FITC | Biolegend | 108406 |
| CD3 | 145-2C11 | PerCPCy5.5 | ThermoFisher | 45-0031-82 |
| CD4 | RM4-5 | PE | BD Biosciences | 553049 |
| CD8 | 53-6.7 | eFluor450 | eBioscience | 48-0081-82 |
| IFNγ | XMG1.2 | FITC | ThermoFisher | 11-7311-82 |
| IL17a | eBio 1787 | APC | ThermoFisher | 17-717781 |
| CD44 | IM7 | APCeFluor780 | ThermoFisher | 47-0441-82 |
| CD62L | MEL-14 | PECy7 | Biolegend | 104418 |

Data S1. (separate file)

Standard target plate and on tissue MS-MS experiments performed at the Johns Hopkins Applied Imaging Mass Spectrometry (AIMS) Core.

Data S2. (separate file)

High mass resolution MALDI imaging experiments performed at the Bruker Daltonics applications laboratory in Billerica, MA.
