# Supplementary material for: CEST MRI and MALDI imaging reveal metabolic alterations in the cervical lymph nodes of EAE mice
Source: J Neuroinflammation. 2022 Jun 3;19:130. doi: 10.1186/s12974-022-02493-z (PMC9164344; doi:10.1186/s12974-022-02493-z)
Supplement: Supplementary file 2 — Additional file 2: Standard target plate and on tissue MS-MS experiments performed at the Johns Hopkins Applied Imaging Mass Spectrometry (AIMS) Core. [file 12974_2022_2493_MOESM2_ESM.pptx]

## Slide 1
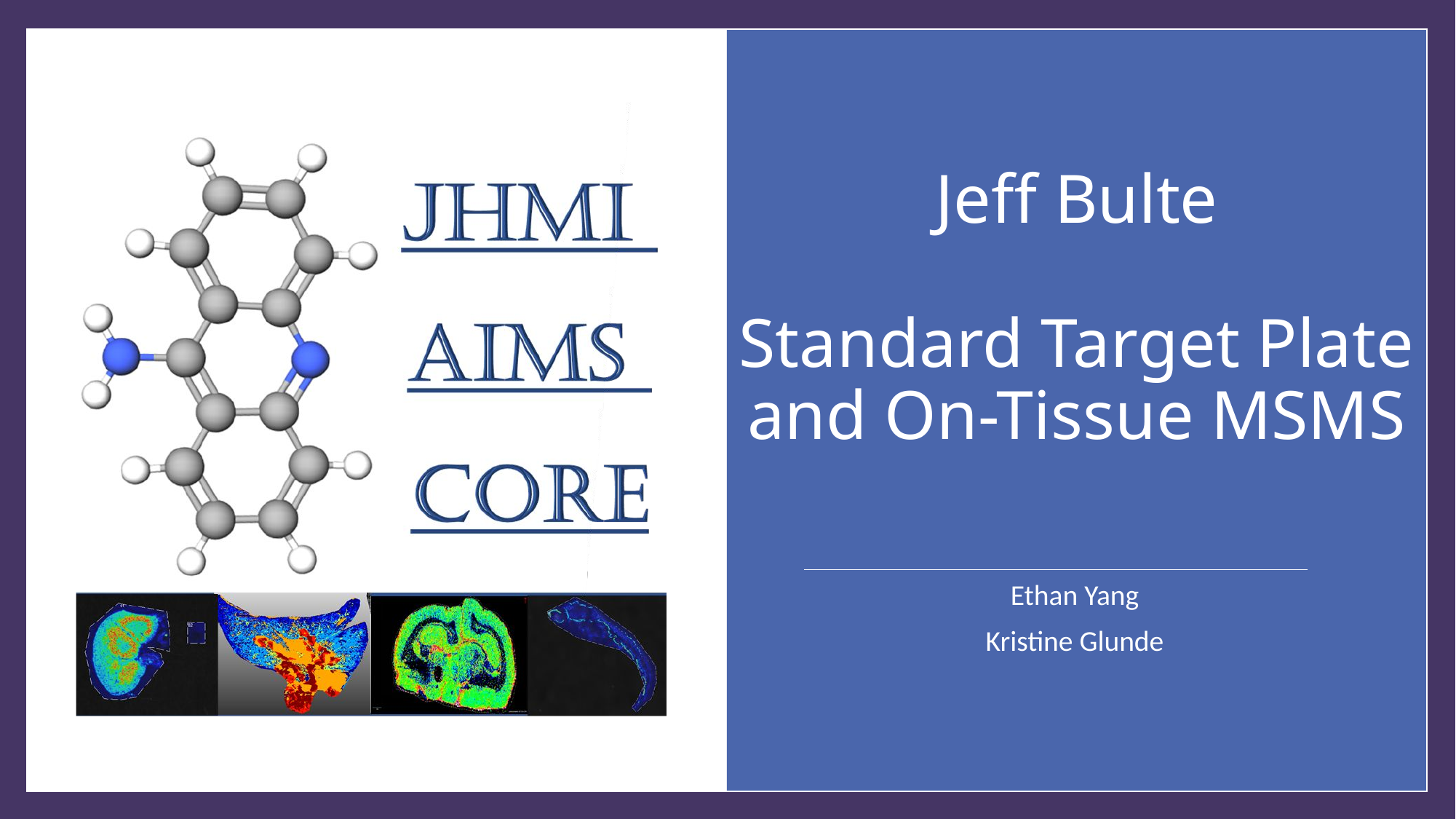

# Jeff Bulte Standard Target Plate and On-Tissue MSMS
Ethan Yang
Kristine Glunde
1

## Slide 2
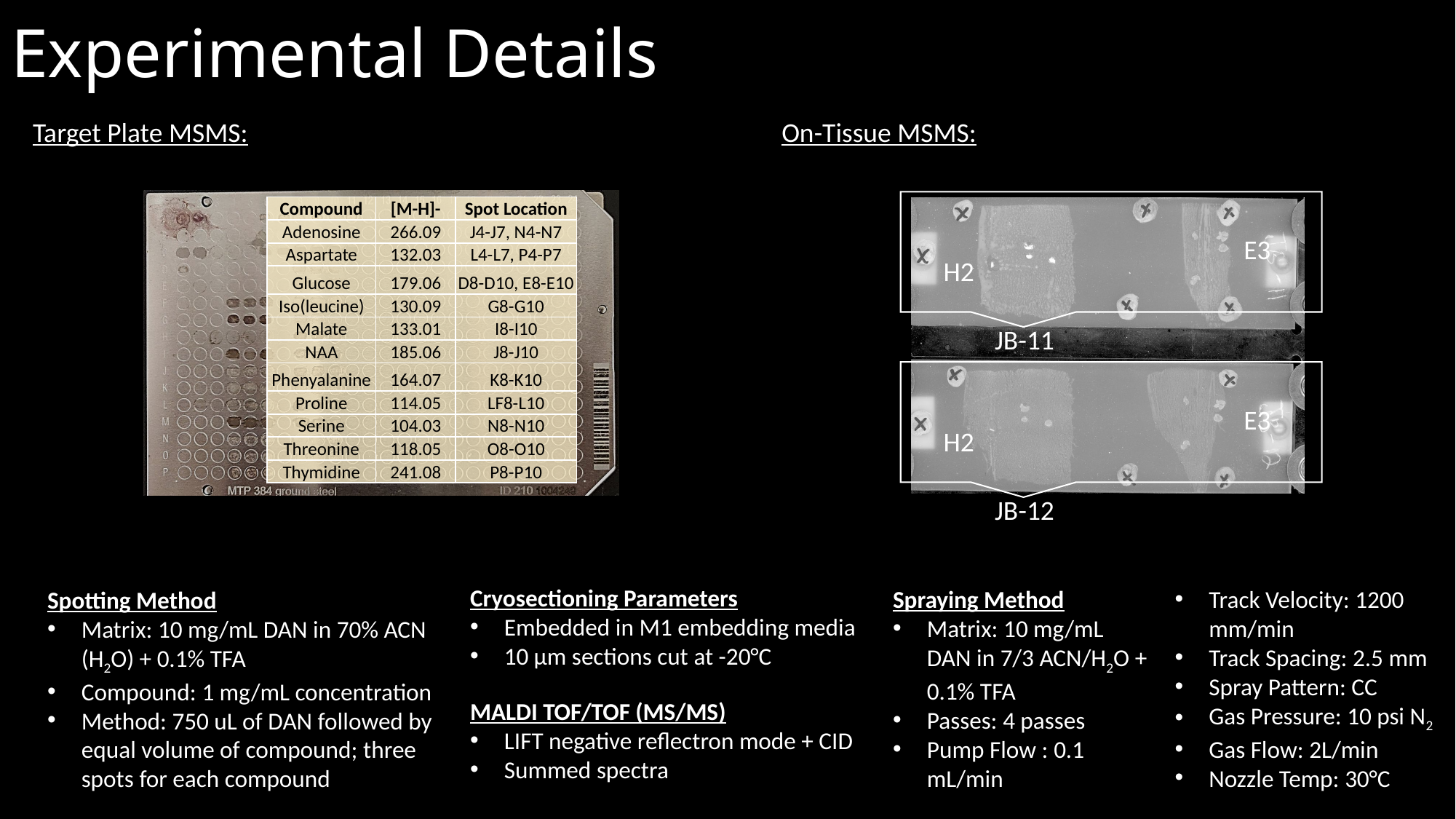

# Experimental Details
Target Plate MSMS:
On-Tissue MSMS:
E3
H2
JB-11
E3
H2
JB-12
| Compound | [M-H]- | Spot Location |
| --- | --- | --- |
| Adenosine | 266.09 | J4-J7, N4-N7 |
| Aspartate | 132.03 | L4-L7, P4-P7 |
| Glucose | 179.06 | D8-D10, E8-E10 |
| Iso(leucine) | 130.09 | G8-G10 |
| Malate | 133.01 | I8-I10 |
| NAA | 185.06 | J8-J10 |
| Phenyalanine | 164.07 | K8-K10 |
| Proline | 114.05 | LF8-L10 |
| Serine | 104.03 | N8-N10 |
| Threonine | 118.05 | O8-O10 |
| Thymidine | 241.08 | P8-P10 |
Cryosectioning Parameters
Embedded in M1 embedding media
10 μm sections cut at -20°C
Spotting Method
Matrix: 10 mg/mL DAN in 70% ACN (H2O) + 0.1% TFA
Compound: 1 mg/mL concentration
Method: 750 uL of DAN followed by equal volume of compound; three spots for each compound
Spraying Method
Matrix: 10 mg/mL DAN in 7/3 ACN/H2O + 0.1% TFA
Passes: 4 passes
Pump Flow : 0.1 mL/min
Track Velocity: 1200 mm/min
Track Spacing: 2.5 mm
Spray Pattern: CC
Gas Pressure: 10 psi N2
Gas Flow: 2L/min
Nozzle Temp: 30°C
MALDI TOF/TOF (MS/MS)
LIFT negative reflectron mode + CID
Summed spectra
2

## Slide 3
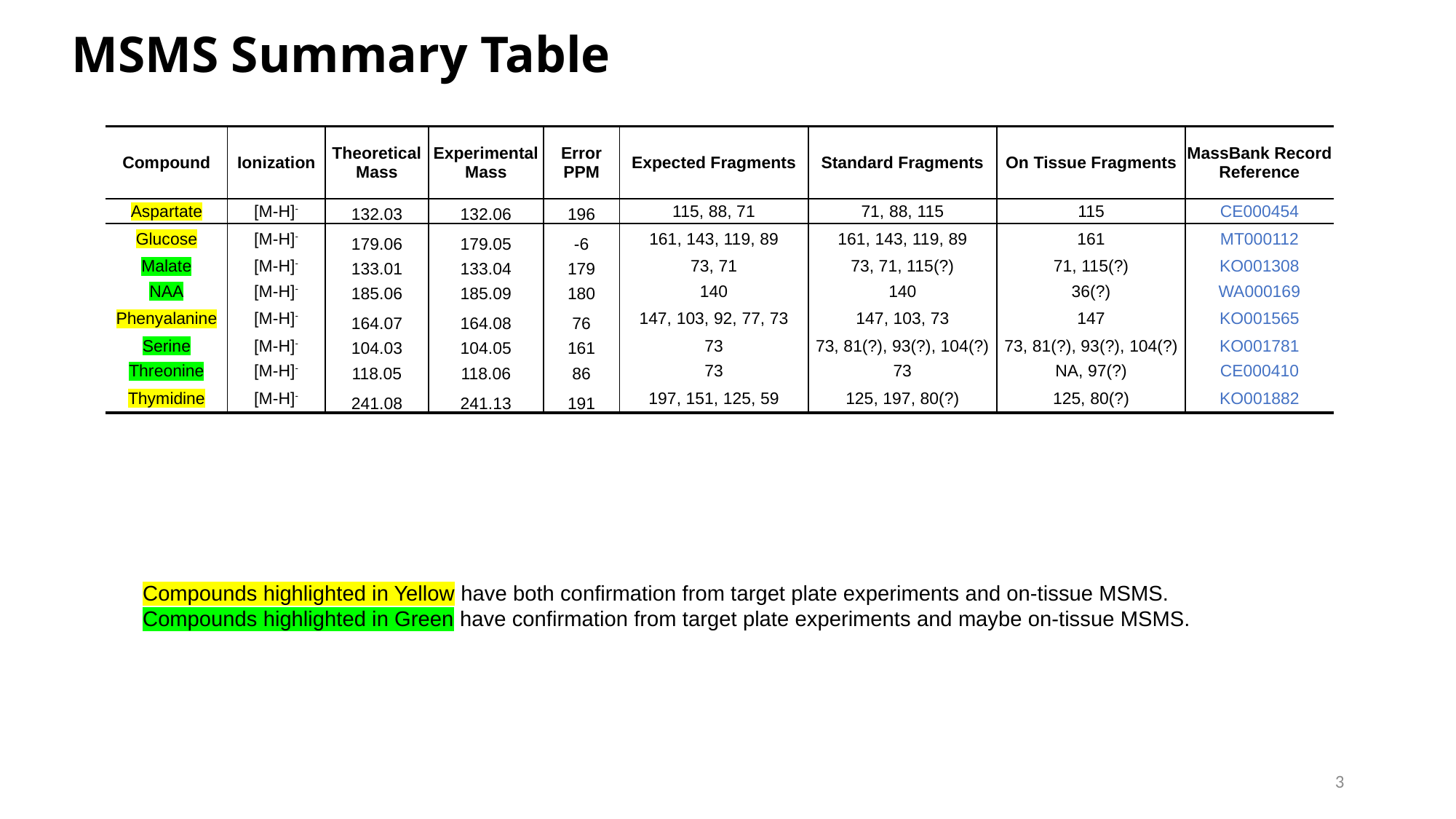

# MSMS Summary Table
| Compound | Ionization | Theoretical Mass | Experimental Mass | Error PPM | Expected Fragments | Standard Fragments | On Tissue Fragments | MassBank Record Reference |
| --- | --- | --- | --- | --- | --- | --- | --- | --- |
| Aspartate | [M-H]- | 132.03 | 132.06 | 196 | 115, 88, 71 | 71, 88, 115 | 115 | CE000454 |
| Glucose | [M-H]- | 179.06 | 179.05 | -6 | 161, 143, 119, 89 | 161, 143, 119, 89 | 161 | MT000112 |
| Malate | [M-H]- | 133.01 | 133.04 | 179 | 73, 71 | 73, 71, 115(?) | 71, 115(?) | KO001308 |
| NAA | [M-H]- | 185.06 | 185.09 | 180 | 140 | 140 | 36(?) | WA000169 |
| Phenyalanine | [M-H]- | 164.07 | 164.08 | 76 | 147, 103, 92, 77, 73 | 147, 103, 73 | 147 | KO001565 |
| Serine | [M-H]- | 104.03 | 104.05 | 161 | 73 | 73, 81(?), 93(?), 104(?) | 73, 81(?), 93(?), 104(?) | KO001781 |
| Threonine | [M-H]- | 118.05 | 118.06 | 86 | 73 | 73 | NA, 97(?) | CE000410 |
| Thymidine | [M-H]- | 241.08 | 241.13 | 191 | 197, 151, 125, 59 | 125, 197, 80(?) | 125, 80(?) | KO001882 |
Compounds highlighted in Yellow have both confirmation from target plate experiments and on-tissue MSMS.
Compounds highlighted in Green have confirmation from target plate experiments and maybe on-tissue MSMS.
3

## Slide 4
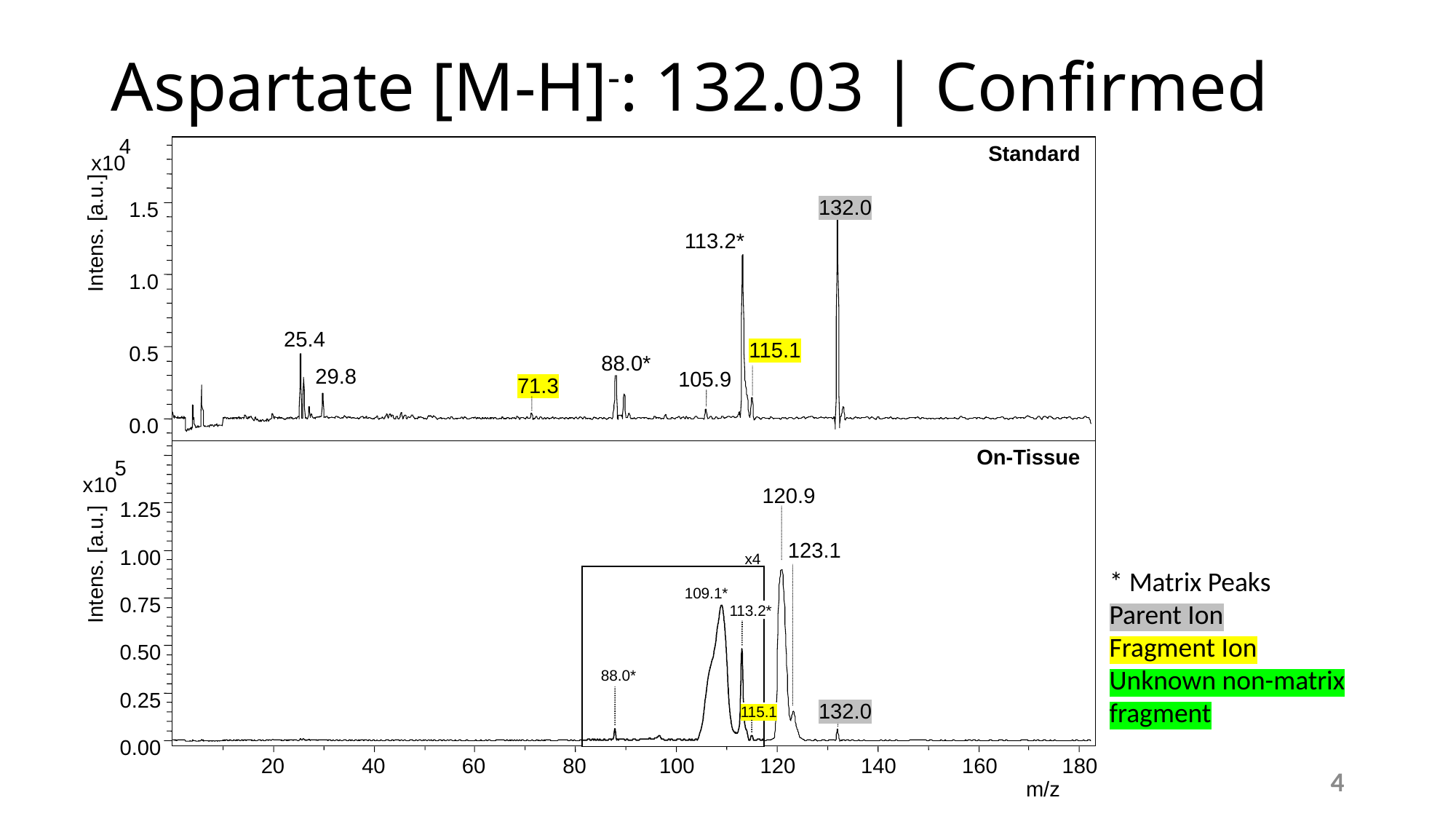

# Aspartate [M-H]-: 132.03 | Confirmed
4
Standard
x10
132.0
1.5
Intens. [a.u.]
1.0
115.1
0.5
88.0*
105.9
71.3
0.0
On-Tissue
5
x10
120.9
1.25
123.1
1.00
Intens. [a.u.]
109.1
0.75
0.50
88.0*
0.25
132.0
0.00
20
40
60
80
100
120
140
160
180
m/z
25.4
29.8
113.2*
x4
109.1*
113.2*
88.0*
115.1
* Matrix Peaks
Parent Ion
Fragment Ion
Unknown non-matrix fragment
4

## Slide 5
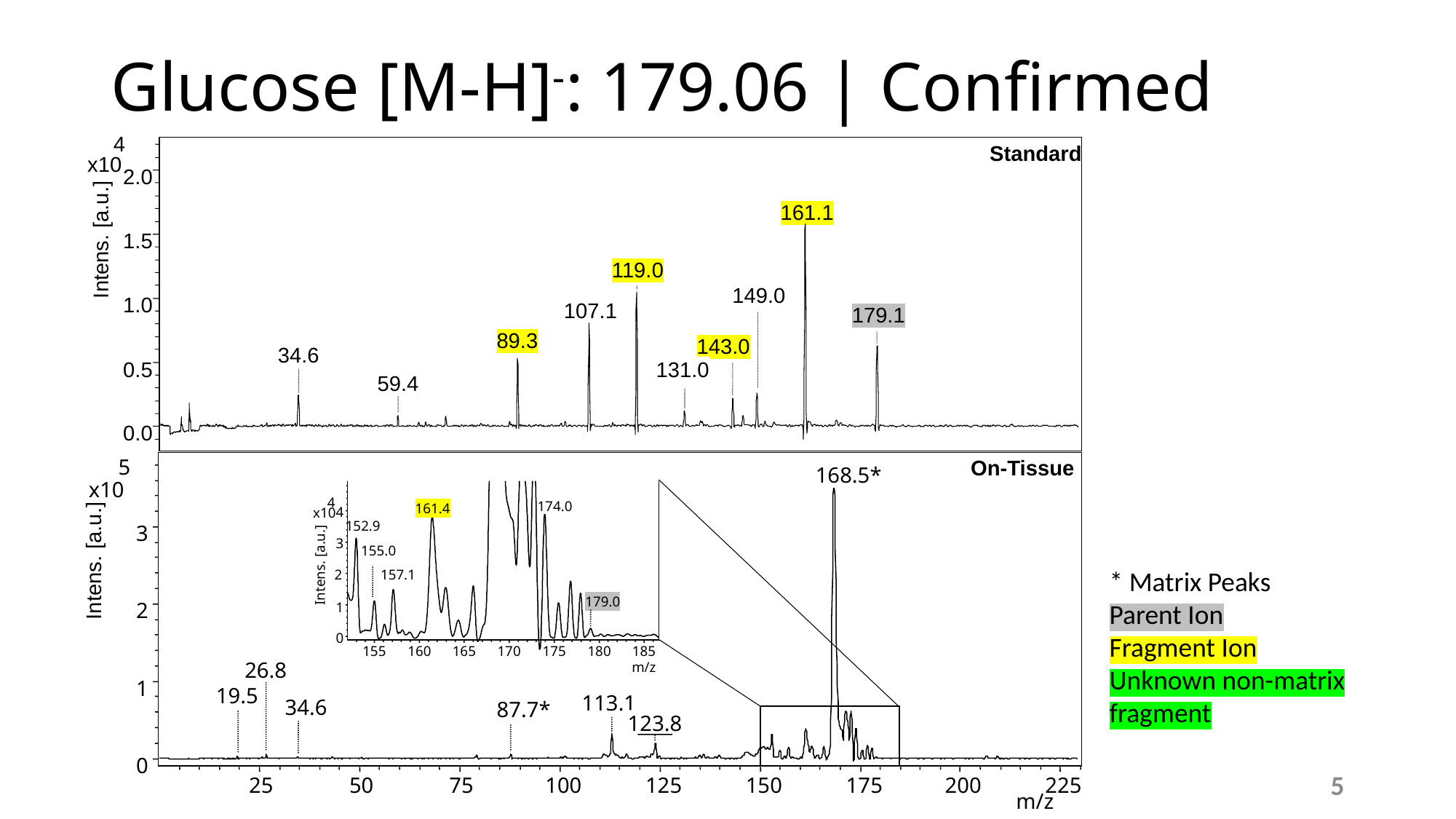

# Glucose [M-H]-: 179.06 | Confirmed
4
Standard
x10
2.0
4
174.0
161.4
4
x10
152.9
3
155.0
Intens. [a.u.]
157.1
2
179.0
1
0
155
160
165
170
175
180
185
m/z
161.1
Intens. [a.u.]
1.5
119.0
149.0
1.0
107.1
179.1
89.3
143.0
34.6
0.5
131.0
59.4
0.0
5
168.5*
x10
3
2
26.8
1
19.5
113.1
34.6
87.7*
123.8
0
25
50
75
100
125
150
175
200
225
m/z
On-Tissue
Intens. [a.u.]
* Matrix Peaks
Parent Ion
Fragment Ion
Unknown non-matrix fragment
5

## Slide 6
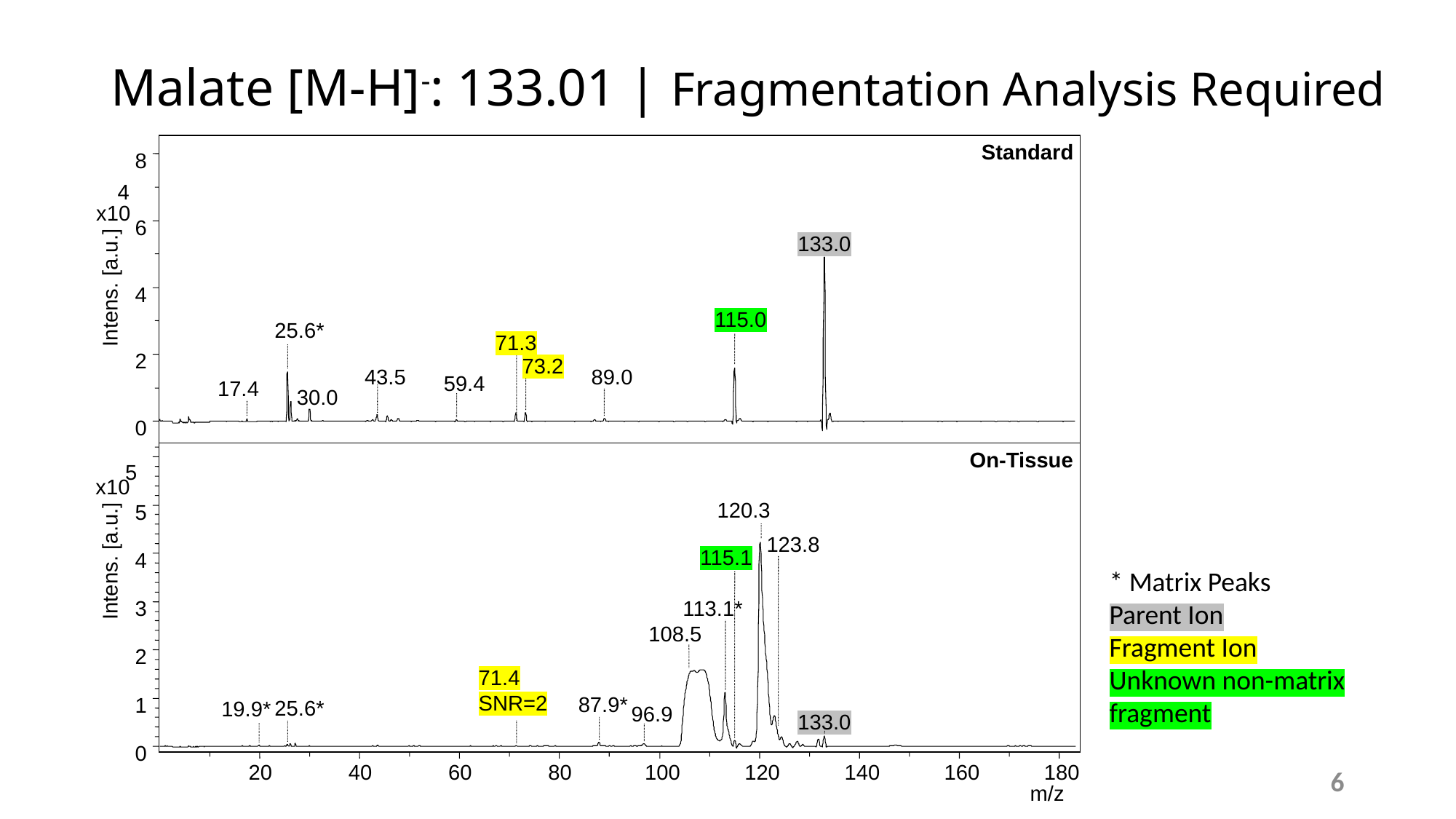

# Malate [M-H]-: 133.01 | Fragmentation Analysis Required
Standard
8
4
x10
6
133.0
Intens. [a.u.]
4
115.0
25.6*
2
73.2
43.5
89.0
59.4
17.4
30.0
0
On-Tissue
5
x10
120.3
5
123.8
115.1
4
Intens. [a.u.]
3
113.1*
108.5
2
71.4
SNR=2
87.9*
1
25.6*
19.9*
96.9
133.0
0
20
40
60
80
100
120
140
160
180
m/z
71.3
* Matrix Peaks
Parent Ion
Fragment Ion
Unknown non-matrix fragment
6

## Slide 7
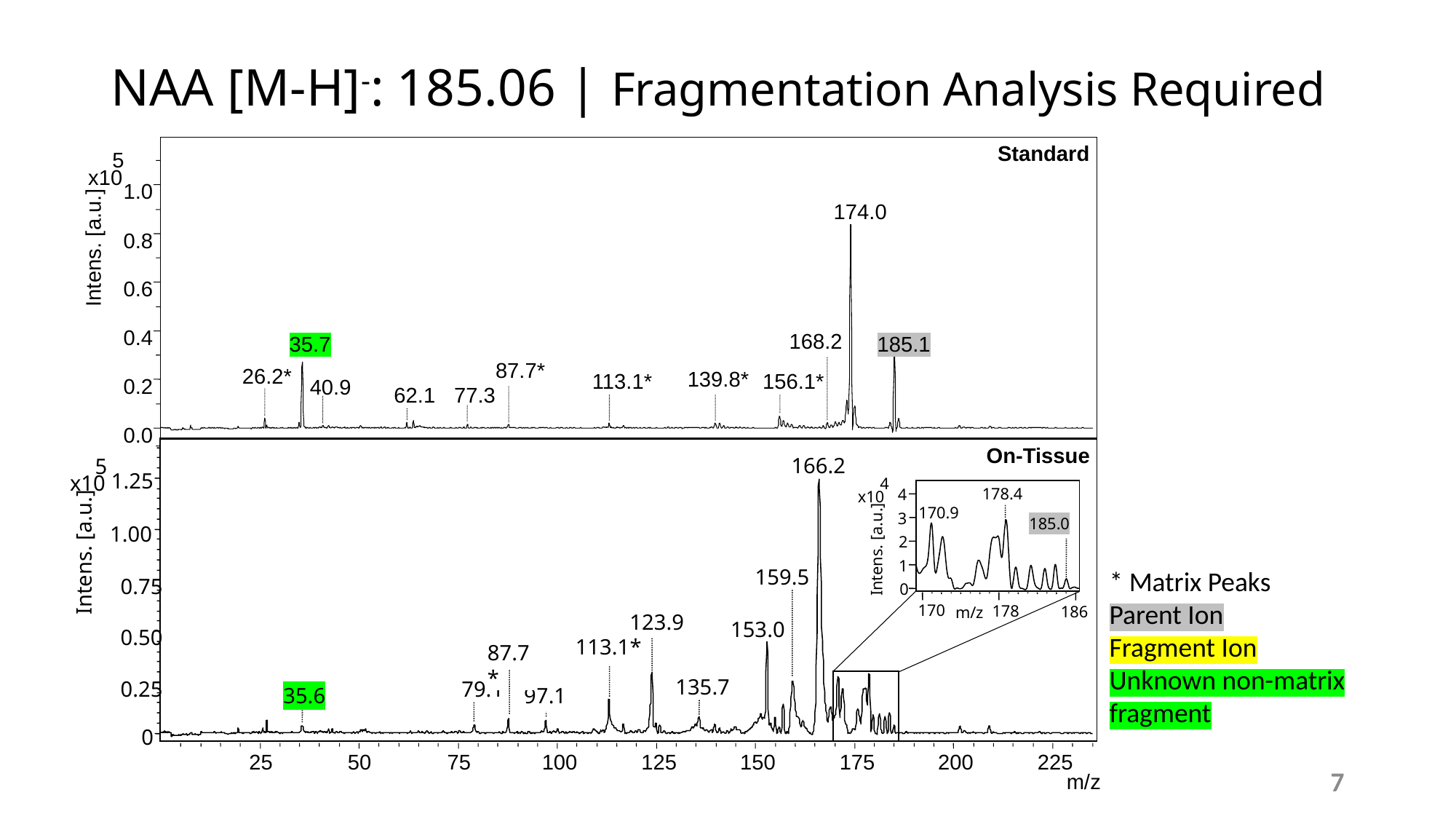

# NAA [M-H]-: 185.06 | Fragmentation Analysis Required
Standard
5
x10
1.0
174.0
0.8
Intens. [a.u.]
0.6
0.4
168.2
35.7
185.1
87.7*
26.2*
139.8*
156.1*
113.1*
0.2
40.9
77.3
62.1
0.0
166.2
5
1.25
x10
1.00
Intens. [a.u.]
159.5
0.75
123.9
153.0
0.50
113.1*
135.7
0.25
79.1
97.1
35.6
0
On-Tissue
4
178.4
4
x10
170.9
3
185.0
Intens. [a.u.]
2
1
0
170
178
186
m/z
87.7*
25
50
75
100
125
150
175
200
225
m/z
* Matrix Peaks
Parent Ion
Fragment Ion
Unknown non-matrix fragment
7

## Slide 8
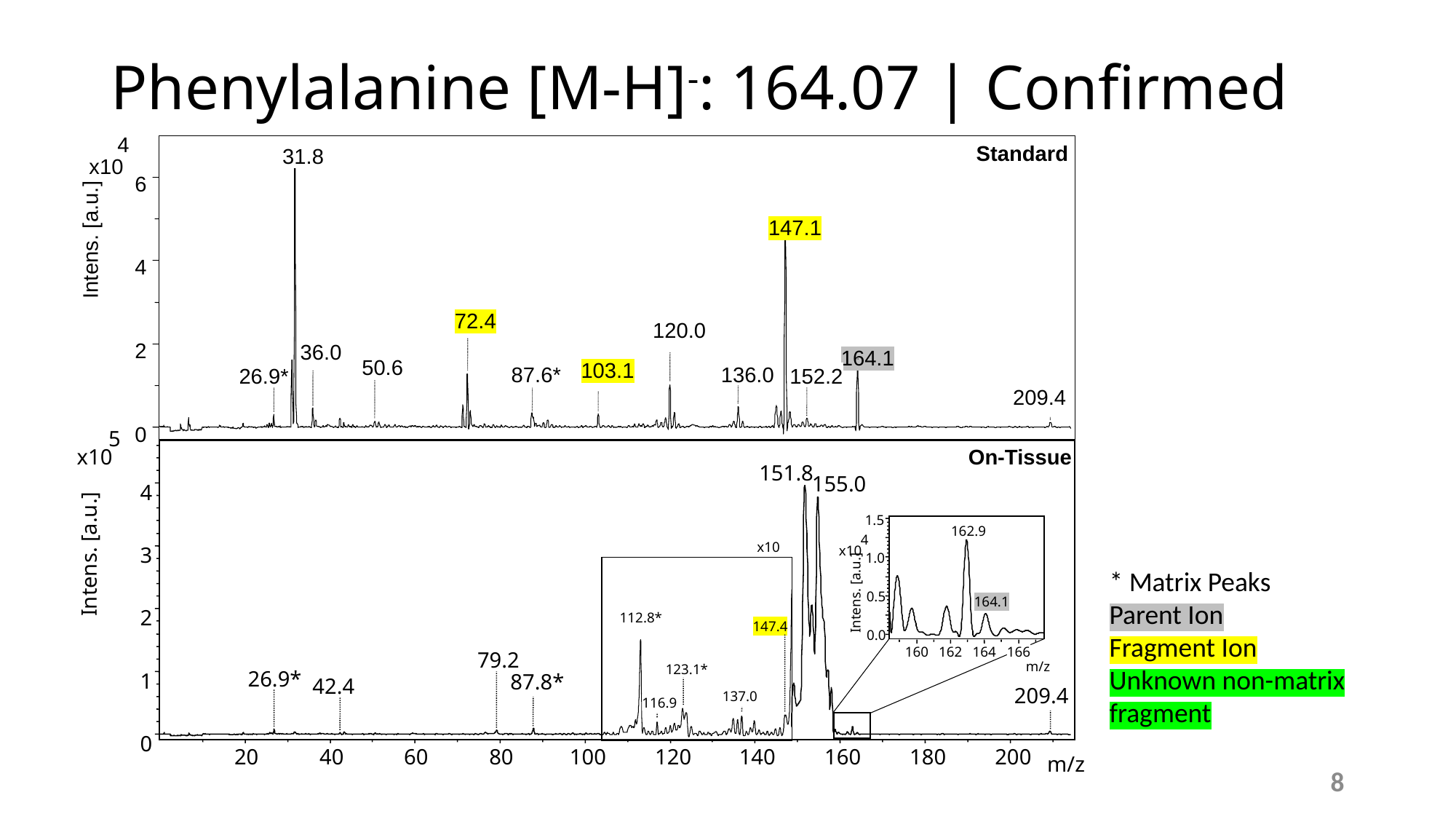

# Phenylalanine [M-H]-: 164.07 | Confirmed
4
Standard
31.8
x10
6
147.1
Intens. [a.u.]
4
72.4
120.0
2
36.0
164.1
50.6
103.1
87.6*
136.0
26.9*
152.2
209.4
0
5
x10
151.8
155.0
4
3
Intens. [a.u.]
2
79.2
26.9*
1
87.8*
42.4
137.0
209.4
123.1*
0
20
40
60
80
100
120
140
160
180
200
m/z
On-Tissue
1.5
162.9
4
x10
1.0
Intens. [a.u.]
0.5
164.1
0.0
160
162
164
166
m/z
x10
112.8*
147.4
123.1*
137.0
116.9
* Matrix Peaks
Parent Ion
Fragment Ion
Unknown non-matrix fragment
8

## Slide 9
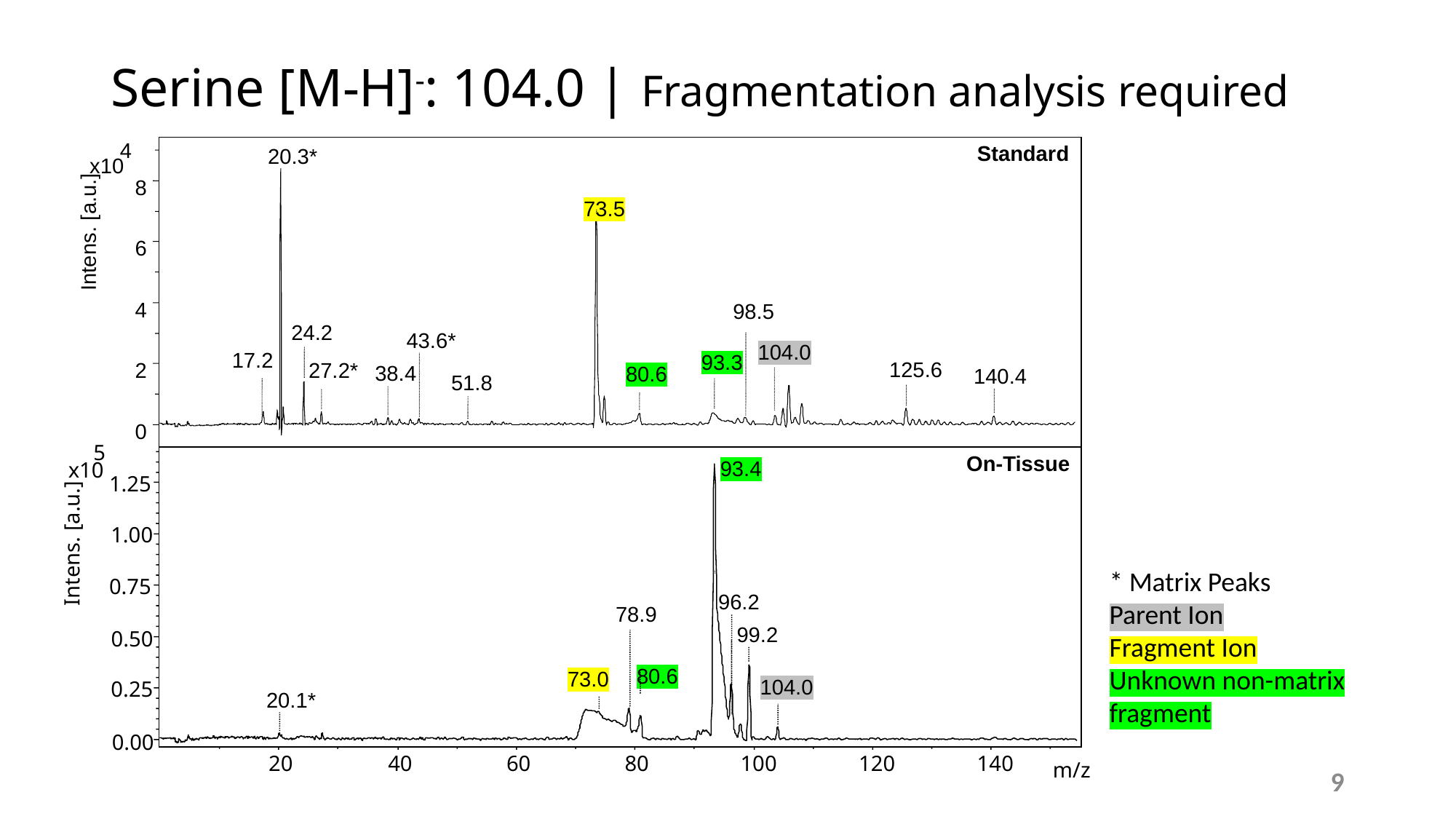

# Serine [M-H]-: 104.0 | Fragmentation analysis required
4
Standard
20.3*
x10
8
73.5
Intens. [a.u.]
6
4
98.5
24.2
43.6*
104.0
17.2
93.3
125.6
2
27.2*
38.4
80.6
140.4
51.8
0
5
x10
1.25
1.00
Intens. [a.u.]
0.75
0.50
0.25
0.00
20
40
60
80
100
120
140
m/z
On-Tissue
93.4
* Matrix Peaks
Parent Ion
Fragment Ion
Unknown non-matrix fragment
96.2
78.9
99.2
80.6
73.0
104.0
20.1*
9

## Slide 10
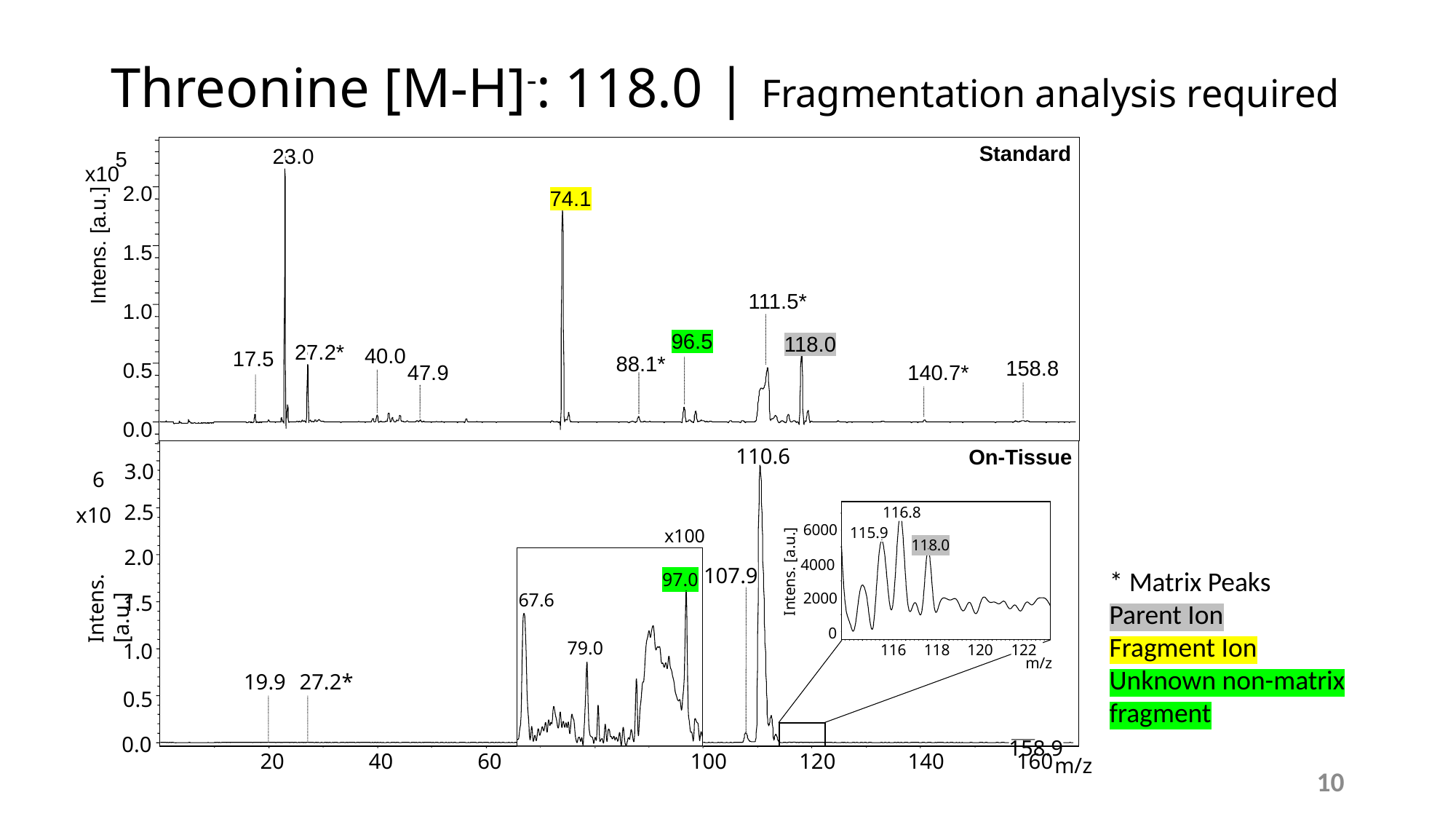

# Threonine [M-H]-: 118.0 | Fragmentation analysis required
Standard
23.0
5
x10
2.0
74.1
Intens. [a.u.]
1.5
111.5*
1.0
96.5
118.0
27.2*
40.0
17.5
88.1*
158.8
0.5
140.7*
47.9
0.0
110.6
3.0
6
2.5
x10
2.0
107.9
Intens. [a.u.]
1.5
1.0
27.2*
19.9
0.5
0.0
158.9
20
40
60
100
120
140
160
m/z
On-Tissue
116.8
6000
115.9
118.0
4000
Intens. [a.u.]
2000
0
116
118
120
122
m/z
x100
97.0
67.6
79.0
* Matrix Peaks
Parent Ion
Fragment Ion
Unknown non-matrix fragment
10

## Slide 11
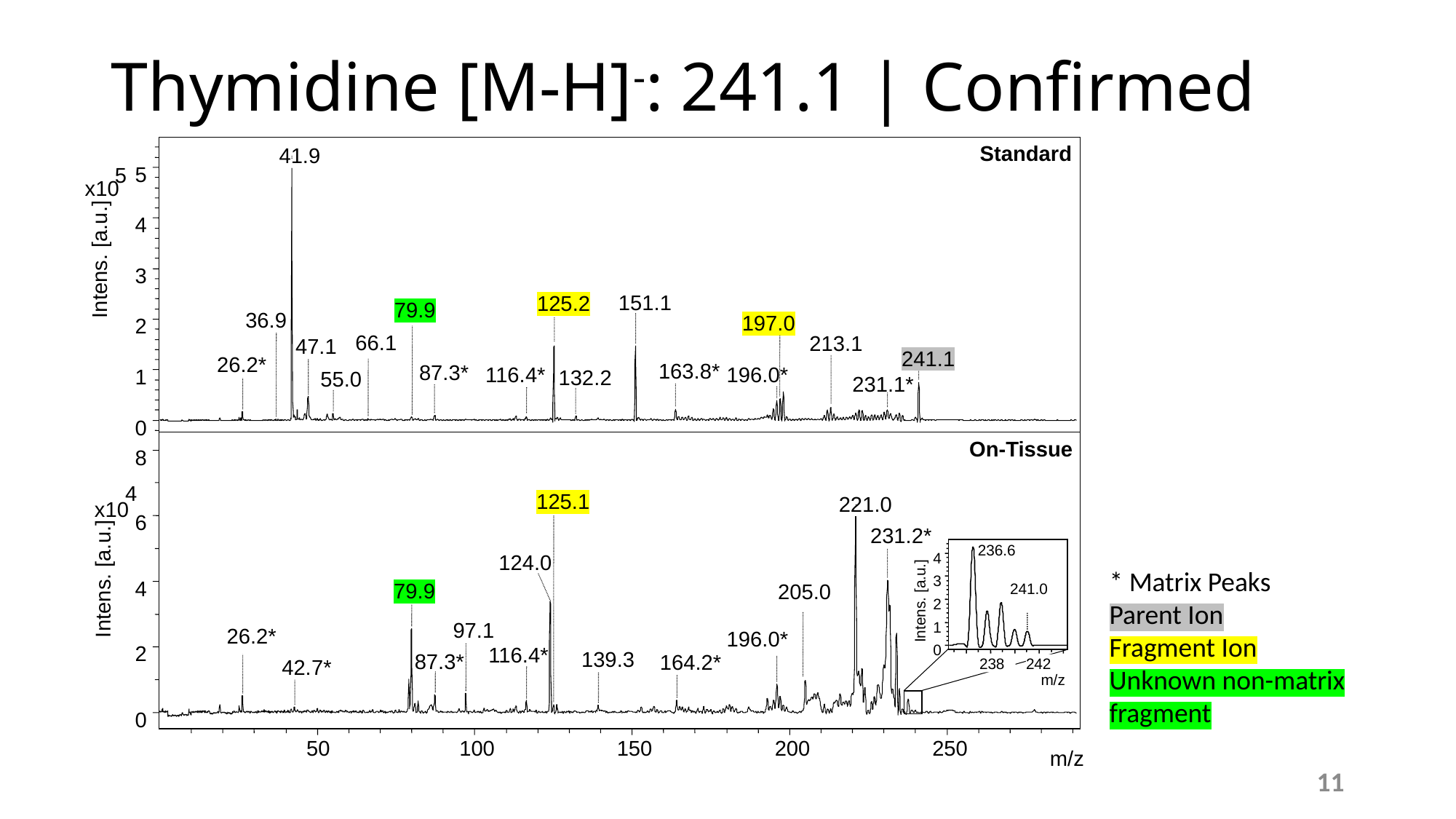

# Thymidine [M-H]-: 241.1 | Confirmed
Standard
41.9
5
5
x10
4
Intens. [a.u.]
3
151.1
125.2
36.9
2
66.1
213.1
47.1
241.1
26.2*
163.8*
87.3*
116.4*
196.0*
1
132.2
55.0
231.1*
0
On-Tissue
8
4
125.1
221.0
x10
6
231.2*
Intens. [a.u.]
4
79.9
205.0
97.1
26.2*
196.0*
2
116.4*
139.3
87.3*
164.2*
42.7*
0
50
100
150
200
250
m/z
197.0
79.9
124.0
236.6
4
3
241.0
2
Intens. [a.u.]
1
0
238
242
m/z
* Matrix Peaks
Parent Ion
Fragment Ion
Unknown non-matrix fragment
11
