# Supplementary material for: CEST MRI and MALDI imaging reveal metabolic alterations in the cervical lymph nodes of EAE mice
Source: J Neuroinflammation. 2022 Jun 3;19:130. doi: 10.1186/s12974-022-02493-z (PMC9164344; doi:10.1186/s12974-022-02493-z)
Supplement: Supplementary file 3 — Additional file 3: High mass resolution MALDI imaging experiments performed at the Bruker Daltonics applications laboratory in Billerica, MA. [file 12974_2022_2493_MOESM3_ESM.pptx]

## Slide 1
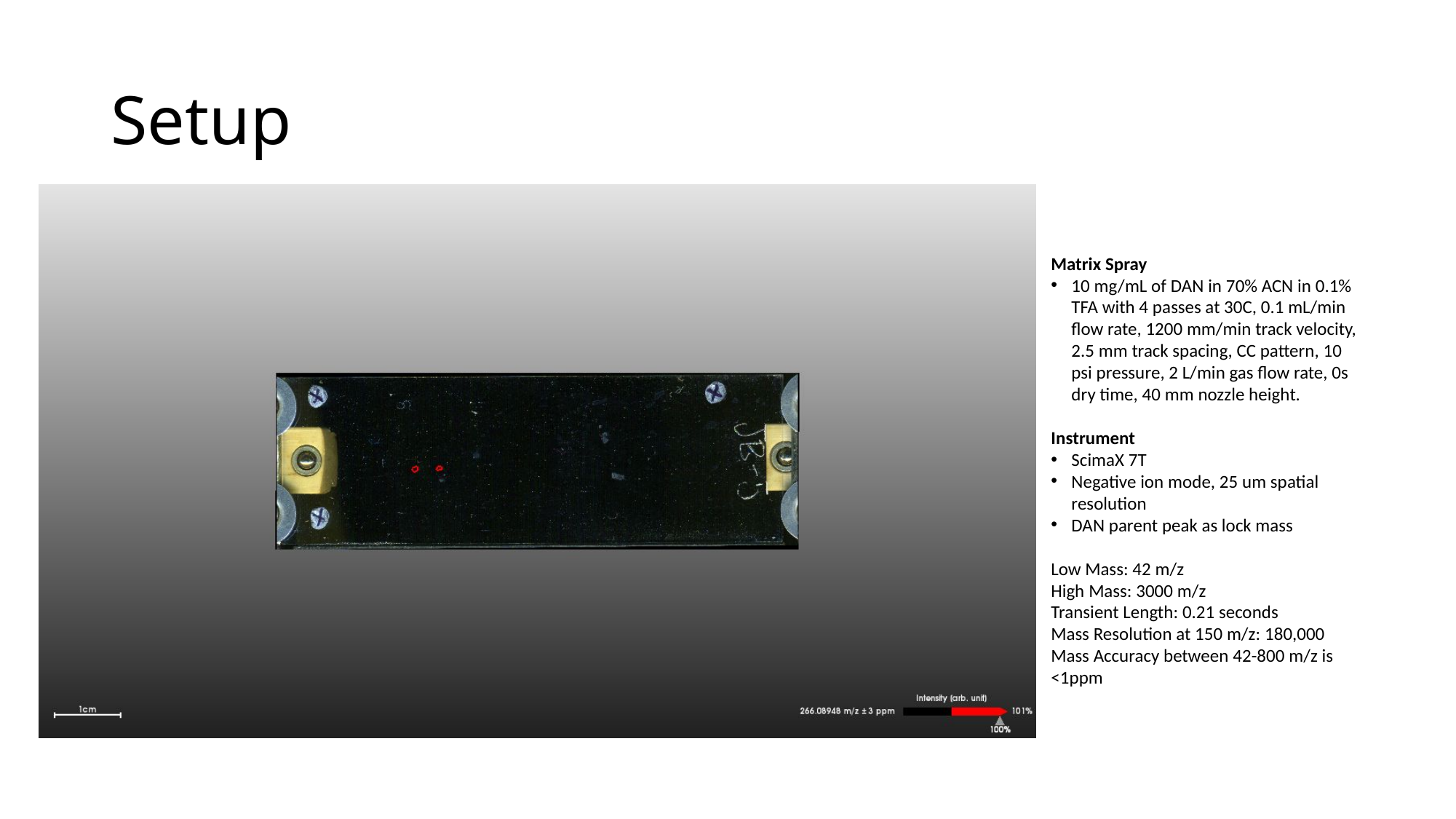

# Setup
Matrix Spray
10 mg/mL of DAN in 70% ACN in 0.1% TFA with 4 passes at 30C, 0.1 mL/min flow rate, 1200 mm/min track velocity, 2.5 mm track spacing, CC pattern, 10 psi pressure, 2 L/min gas flow rate, 0s dry time, 40 mm nozzle height.
Instrument
ScimaX 7T
Negative ion mode, 25 um spatial resolution
DAN parent peak as lock mass
Low Mass: 42 m/z
High Mass: 3000 m/z
Transient Length: 0.21 seconds
Mass Resolution at 150 m/z: 180,000
Mass Accuracy between 42-800 m/z is <1ppm

## Slide 2
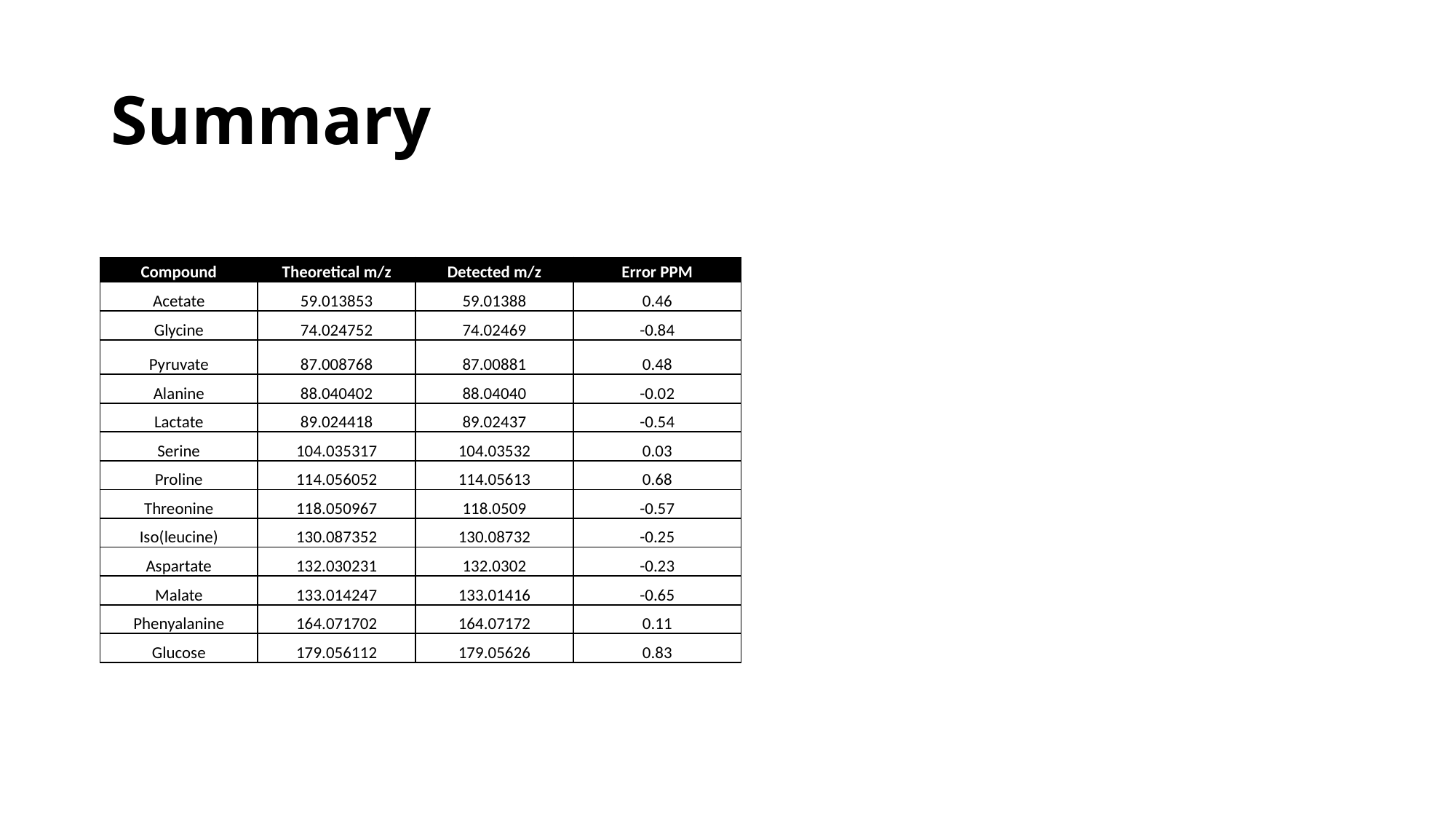

# Summary
| Compound | Theoretical m/z | Detected m/z | Error PPM |
| --- | --- | --- | --- |
| Acetate | 59.013853 | 59.01388 | 0.46 |
| Glycine | 74.024752 | 74.02469 | -0.84 |
| Pyruvate | 87.008768 | 87.00881 | 0.48 |
| Alanine | 88.040402 | 88.04040 | -0.02 |
| Lactate | 89.024418 | 89.02437 | -0.54 |
| Serine | 104.035317 | 104.03532 | 0.03 |
| Proline | 114.056052 | 114.05613 | 0.68 |
| Threonine | 118.050967 | 118.0509 | -0.57 |
| Iso(leucine) | 130.087352 | 130.08732 | -0.25 |
| Aspartate | 132.030231 | 132.0302 | -0.23 |
| Malate | 133.014247 | 133.01416 | -0.65 |
| Phenyalanine | 164.071702 | 164.07172 | 0.11 |
| Glucose | 179.056112 | 179.05626 | 0.83 |

## Slide 3
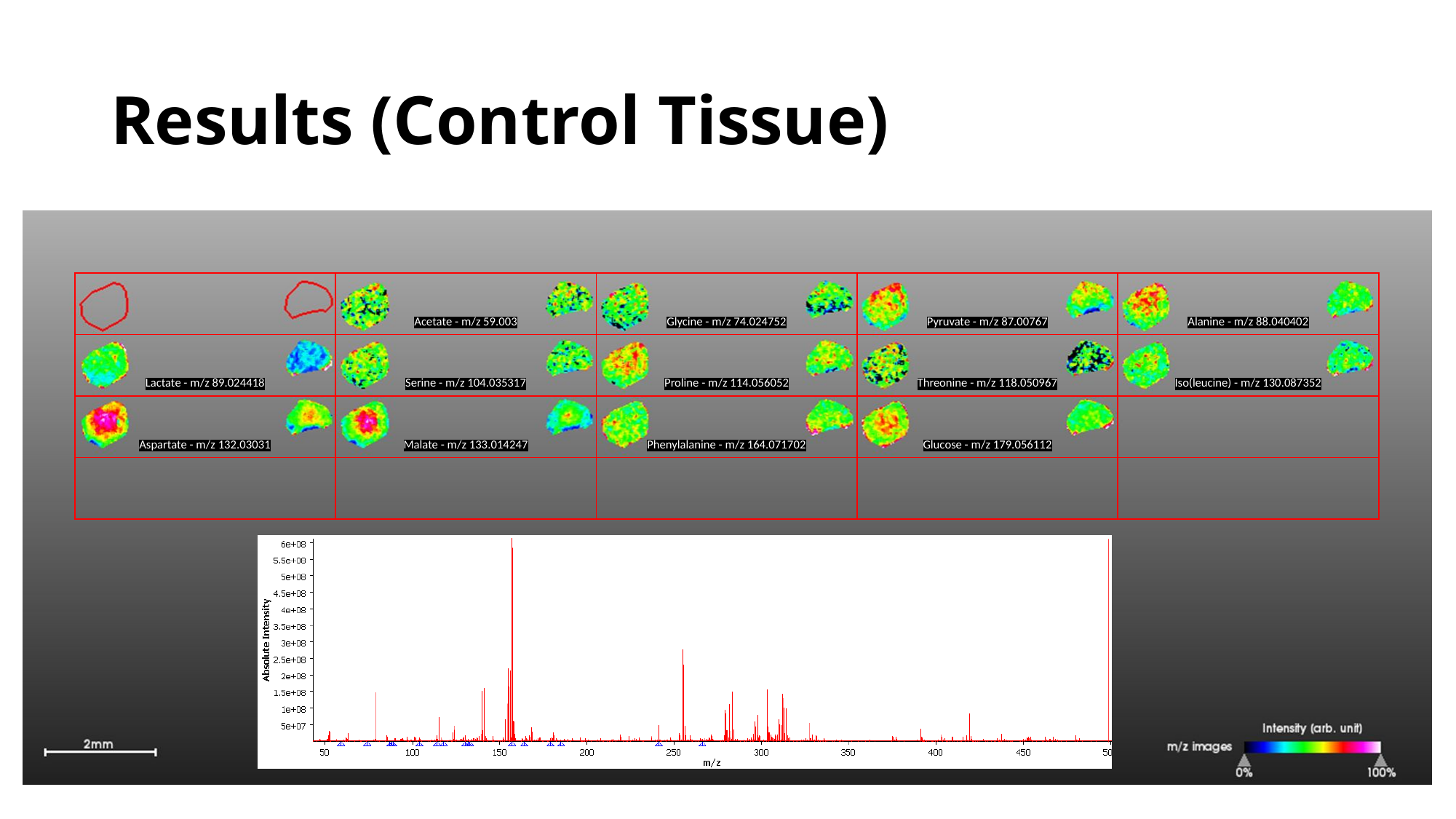

# Results (Control Tissue)
| | Acetate - m/z 59.003 | Glycine - m/z 74.024752 | Pyruvate - m/z 87.00767 | Alanine - m/z 88.040402 |
| --- | --- | --- | --- | --- |
| Lactate - m/z 89.024418 | Serine - m/z 104.035317 | Proline - m/z 114.056052 | Threonine - m/z 118.050967 | Iso(leucine) - m/z 130.087352 |
| Aspartate - m/z 132.03031 | Malate - m/z 133.014247 | Phenylalanine - m/z 164.071702 | Glucose - m/z 179.056112 | |
| | | | | |

## Slide 4
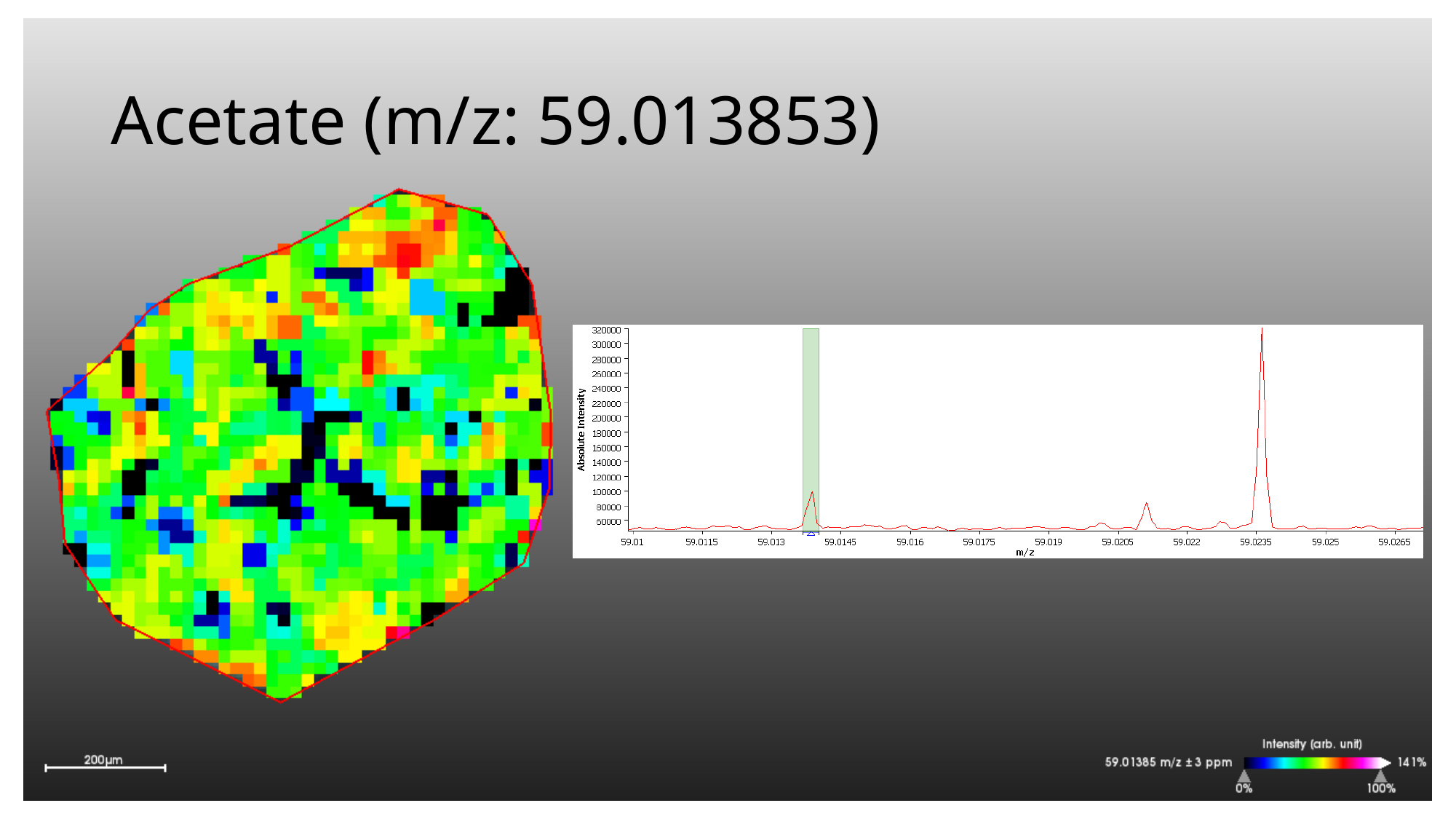

# Acetate (m/z: 59.013853)

## Slide 5
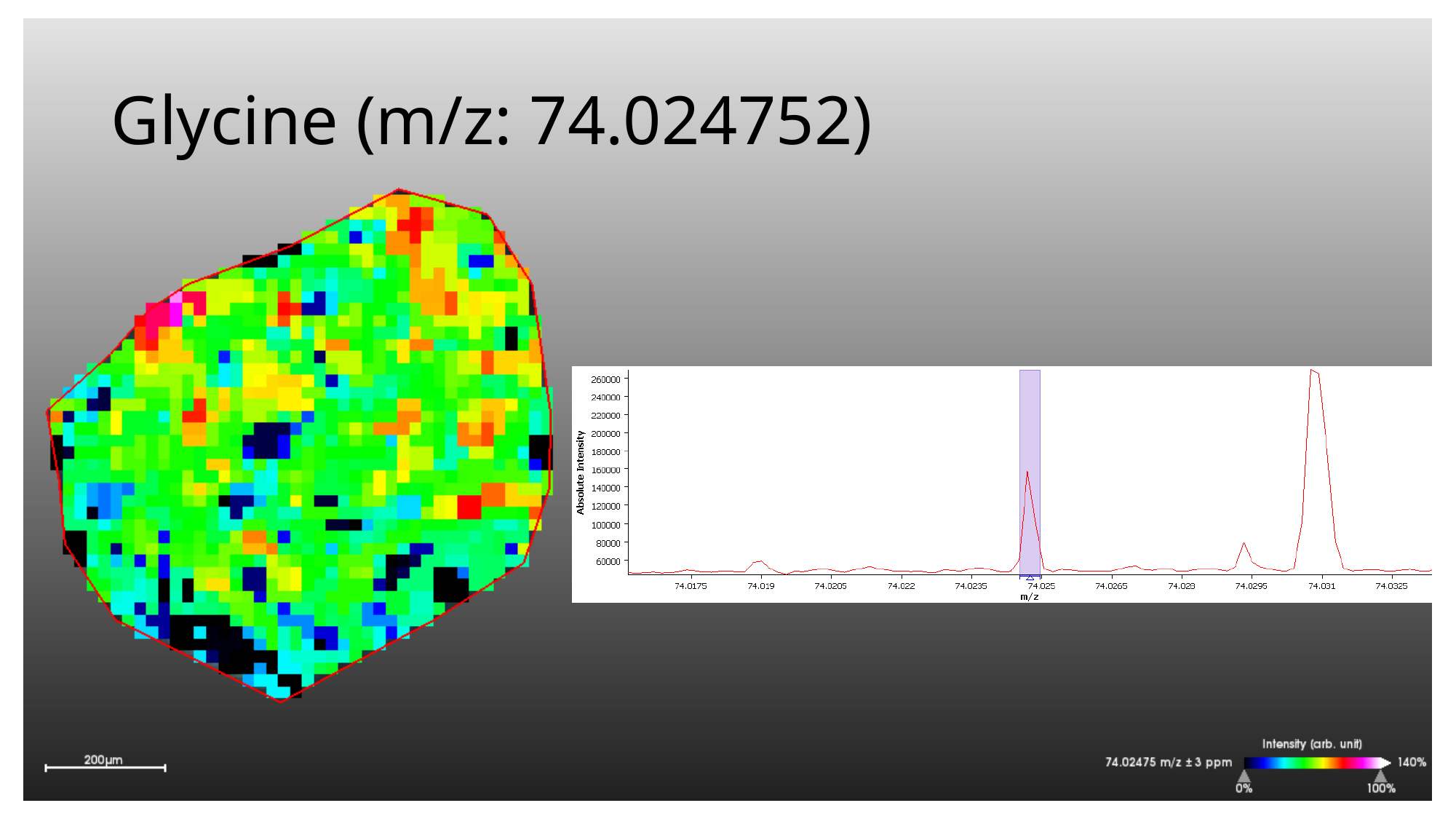

# Glycine (m/z: 74.024752)

## Slide 6
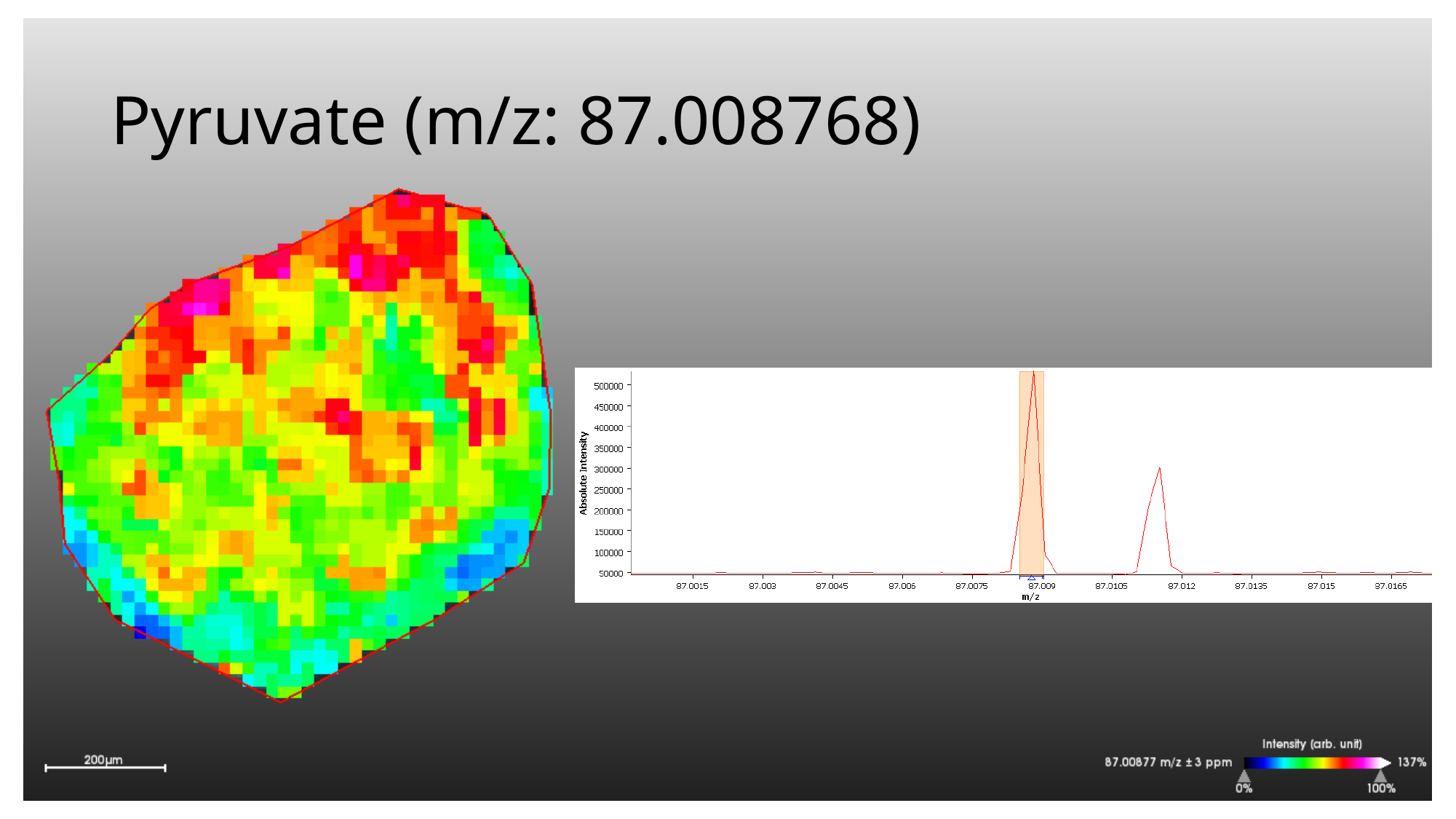

# Pyruvate (m/z: 87.008768)

## Slide 7
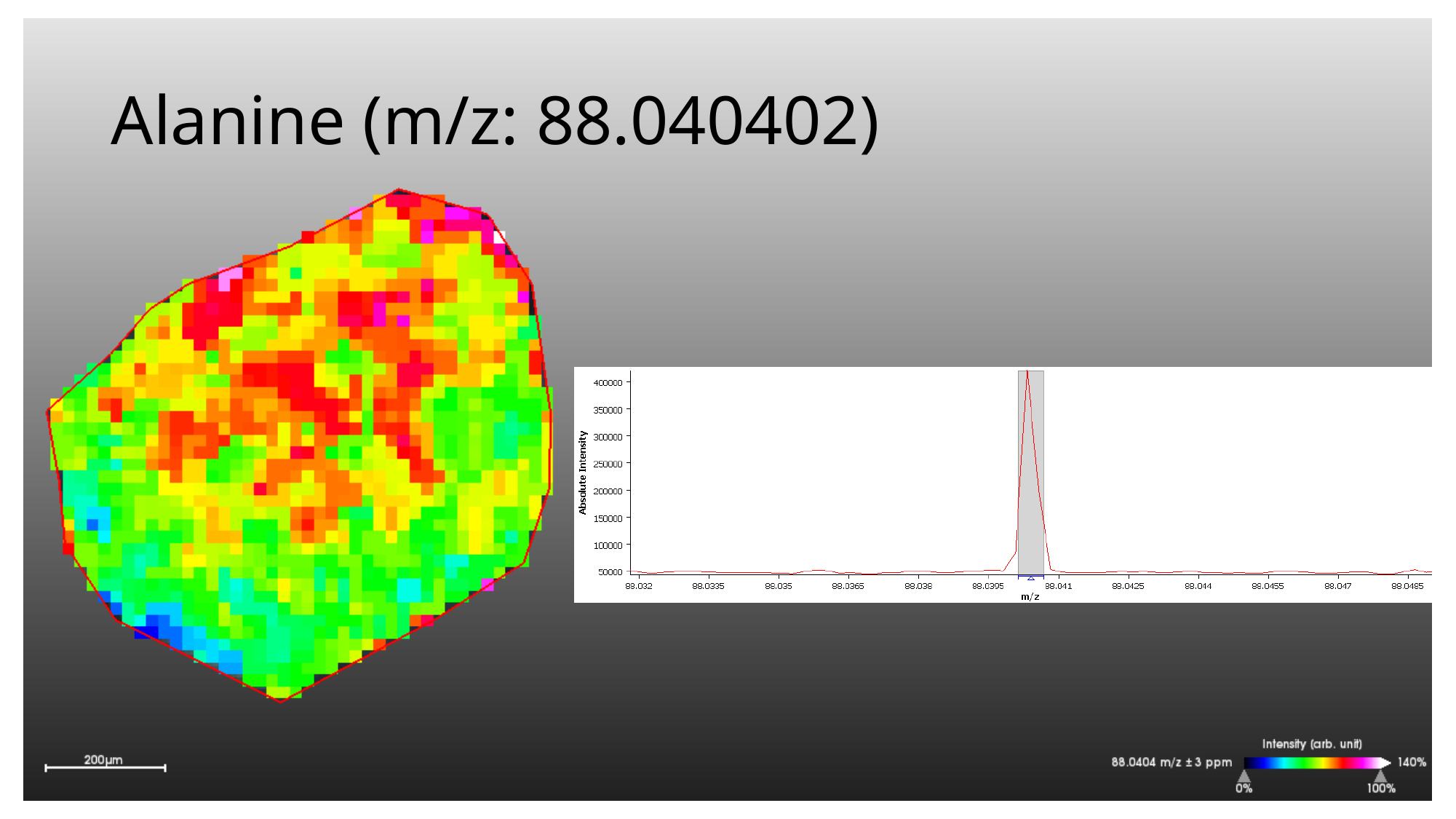

# Alanine (m/z: 88.040402)

## Slide 8
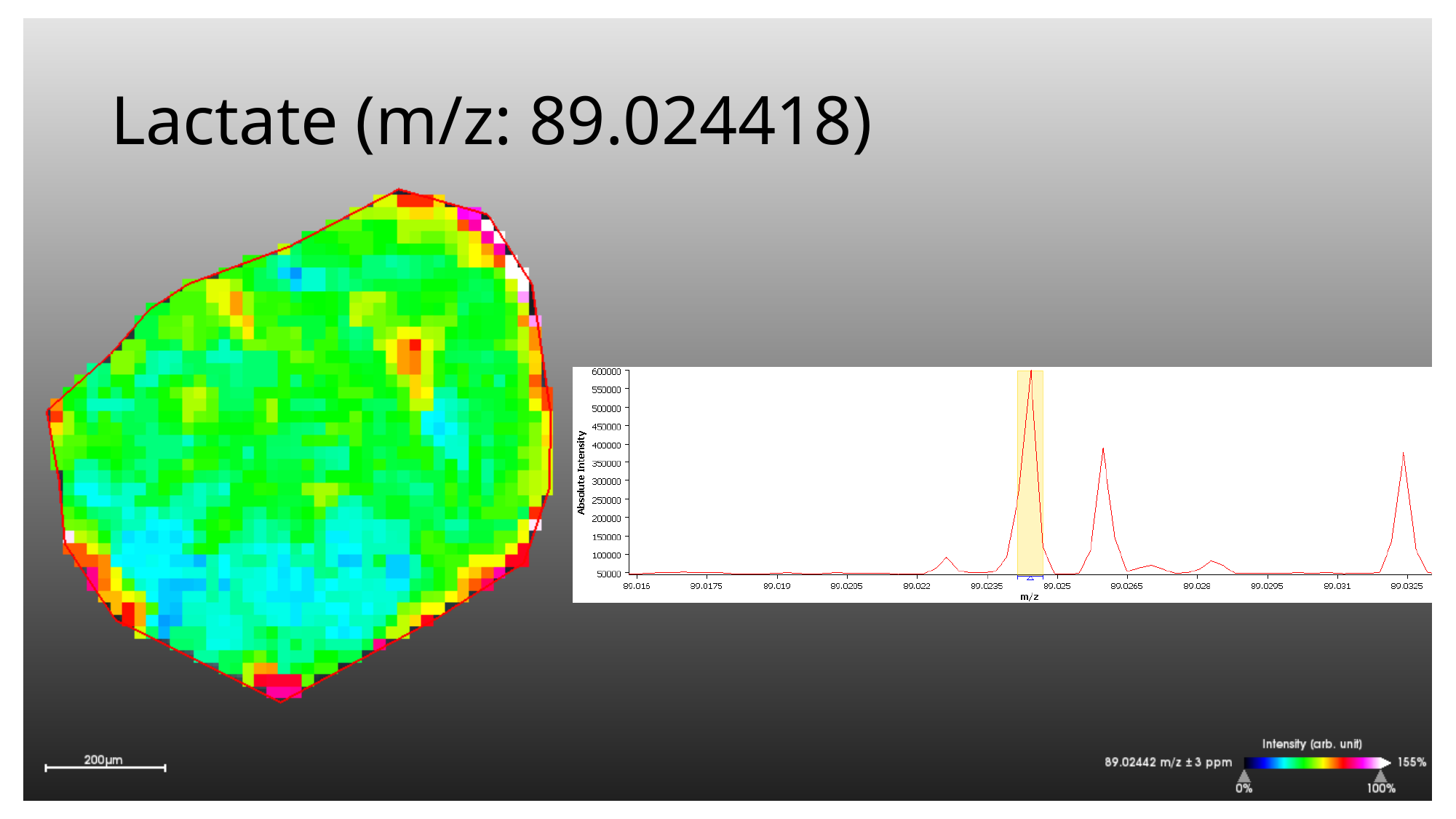

# Lactate (m/z: 89.024418)

## Slide 9
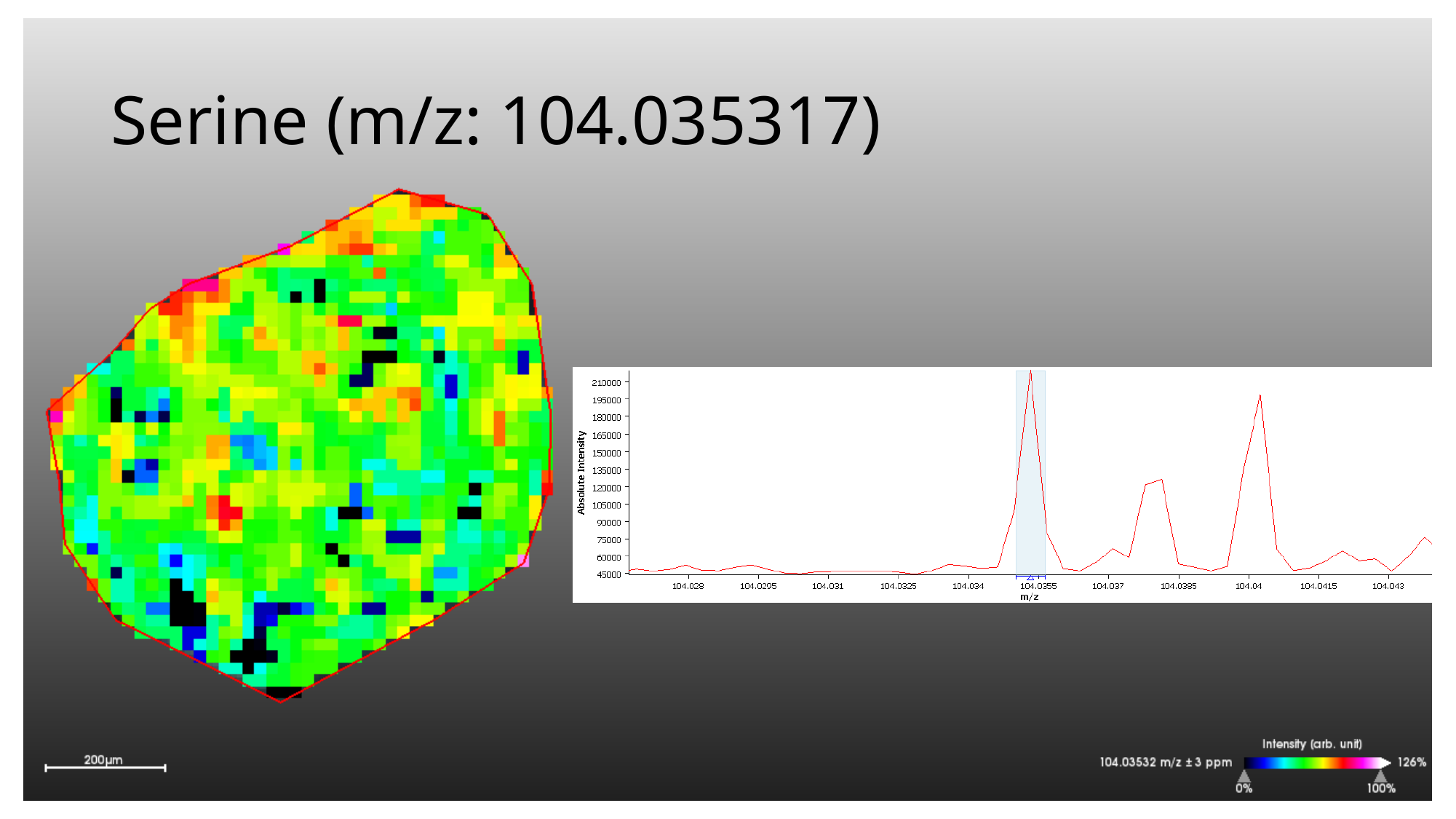

# Serine (m/z: 104.035317)

## Slide 10
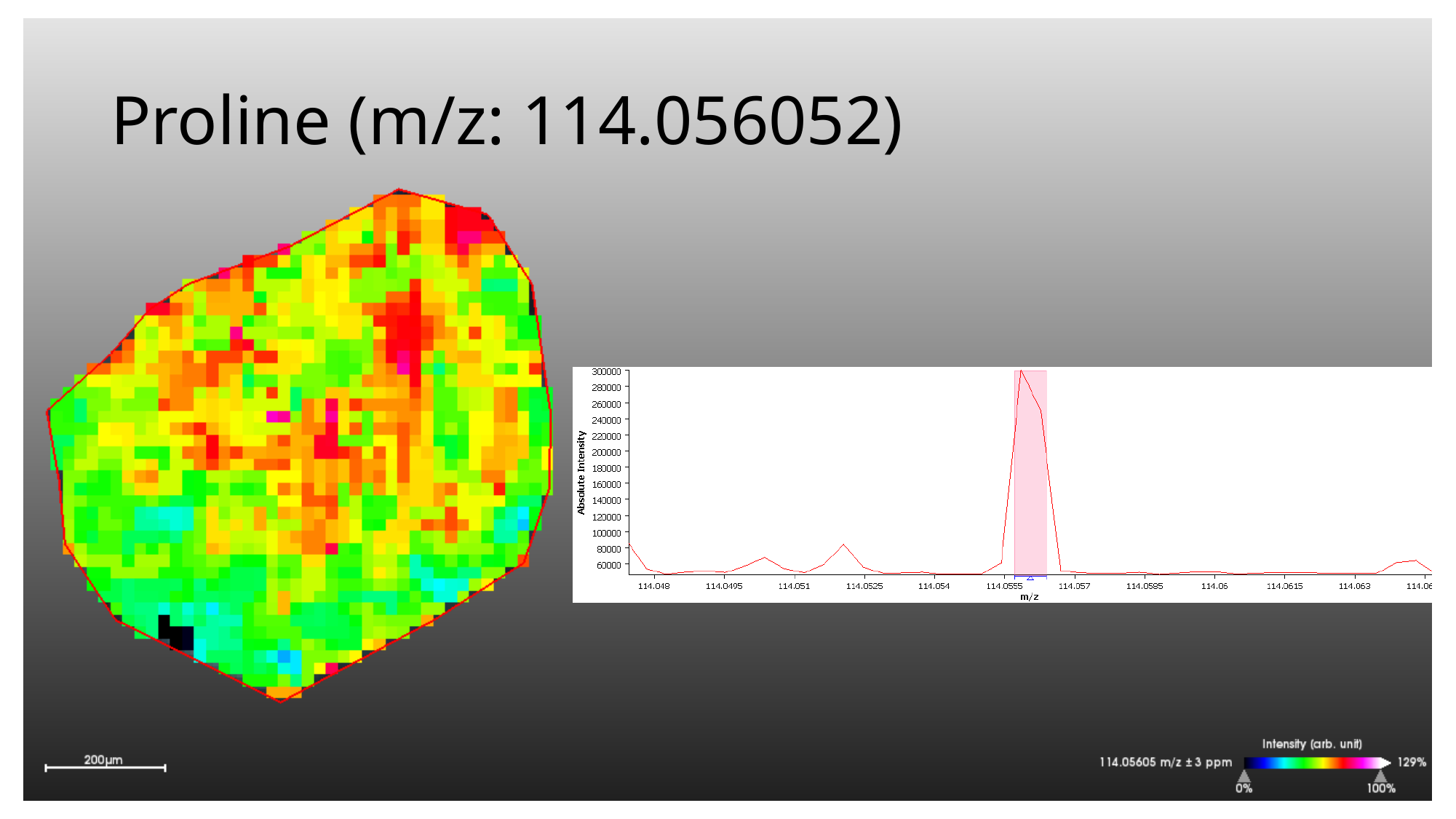

# Proline (m/z: 114.056052)

## Slide 11
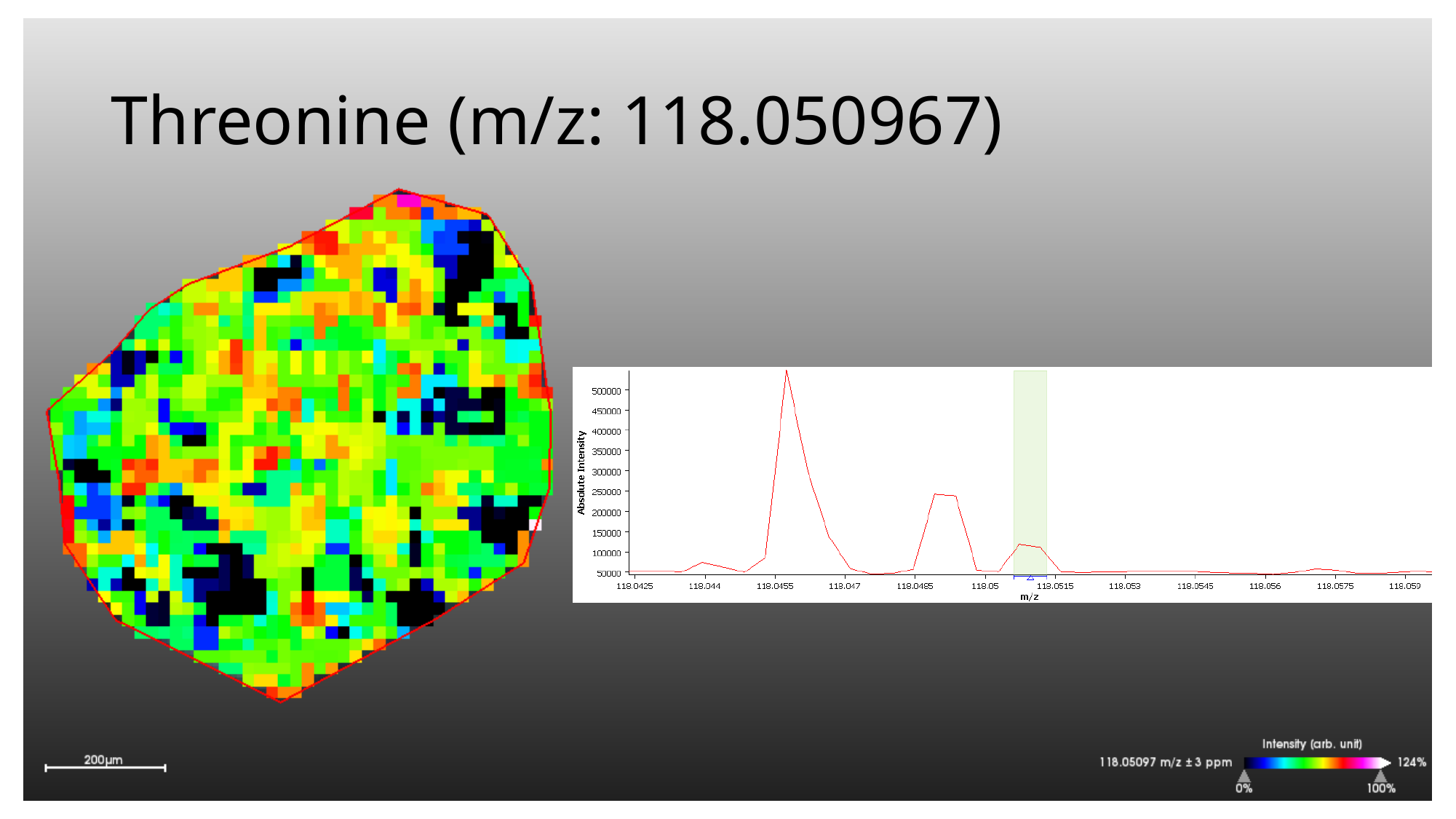

# Threonine (m/z: 118.050967)

## Slide 12
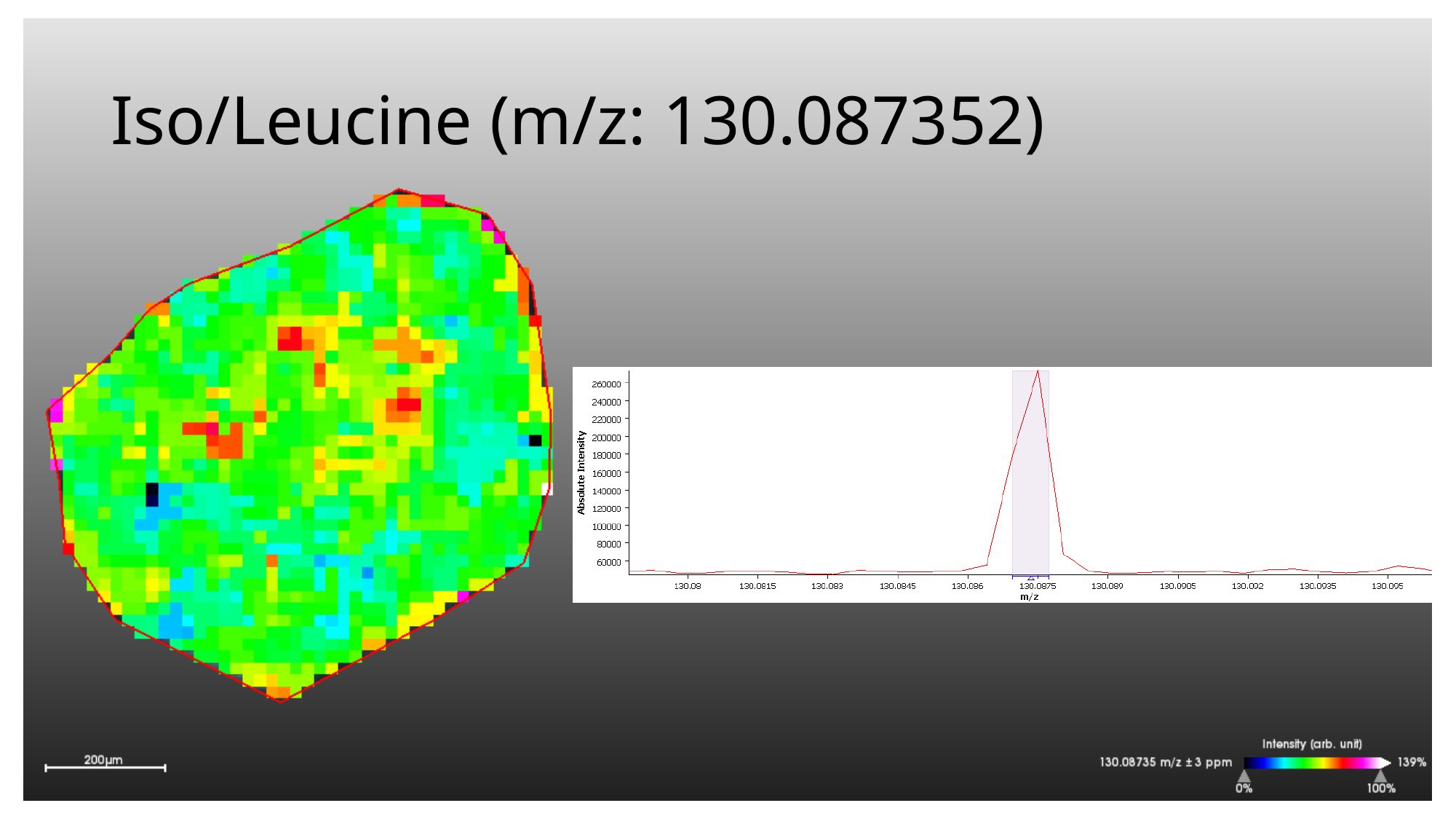

# Iso/Leucine (m/z: 130.087352)

## Slide 13
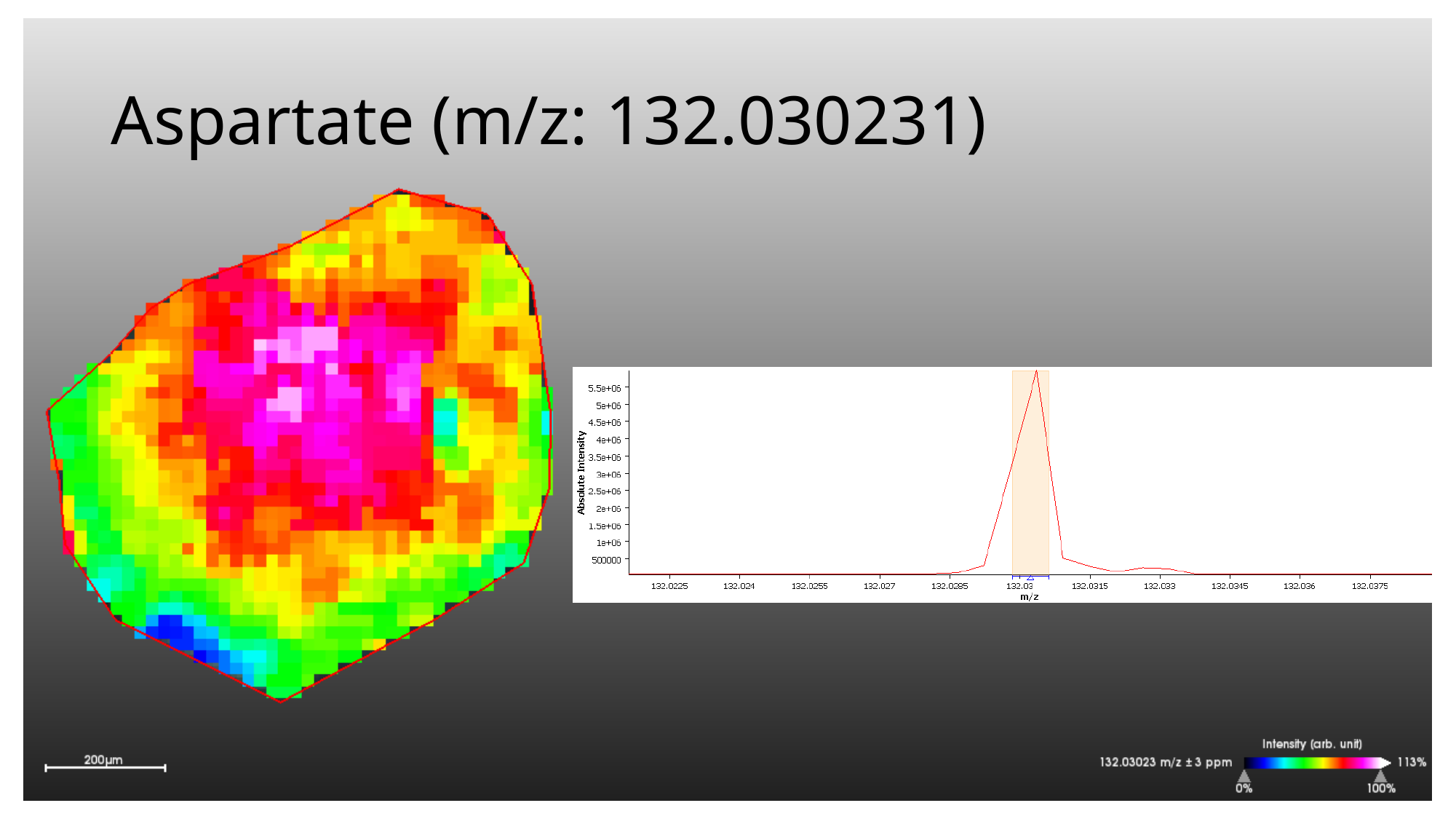

# Aspartate (m/z: 132.030231)

## Slide 14
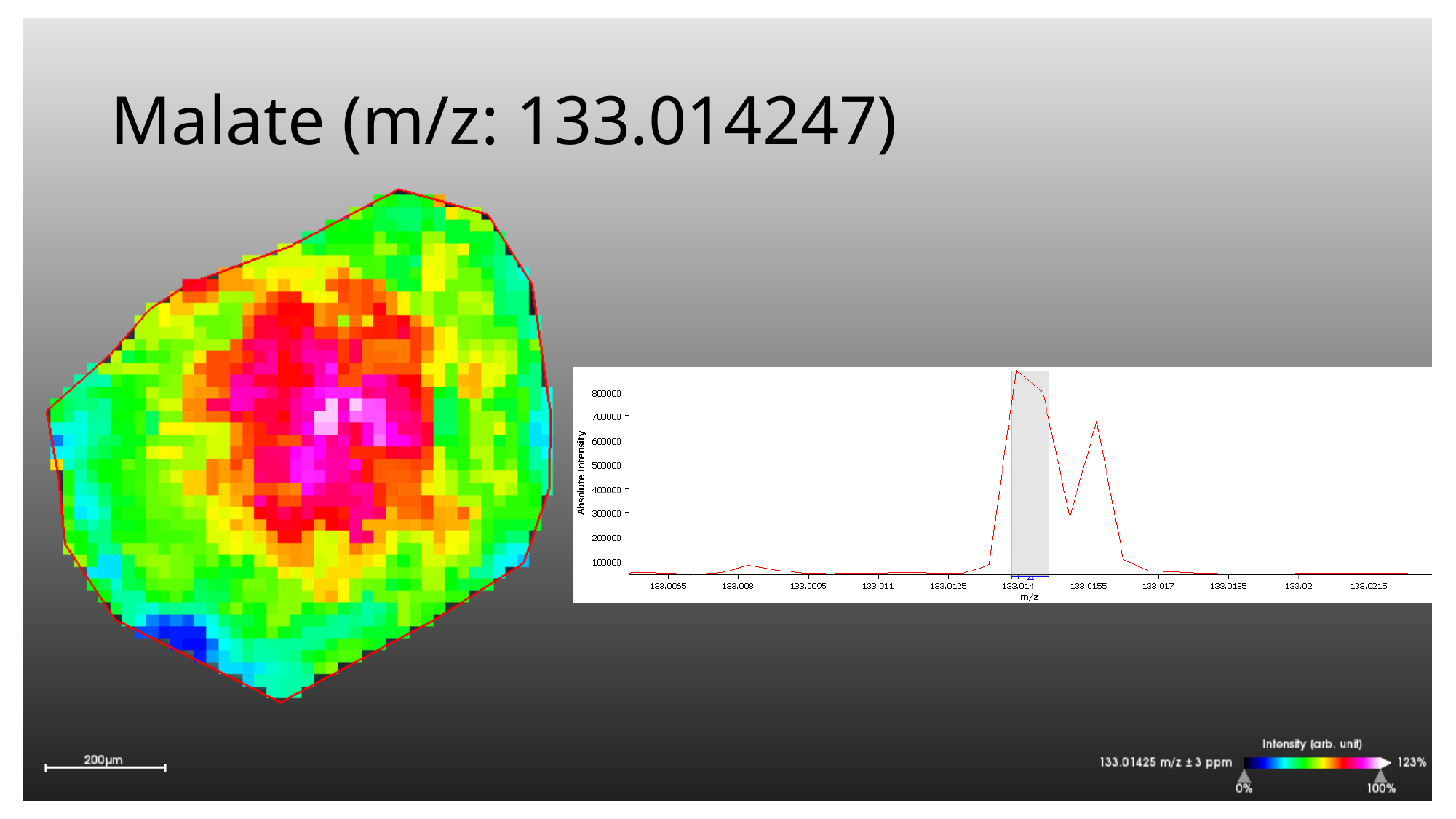

# Malate (m/z: 133.014247)

## Slide 15
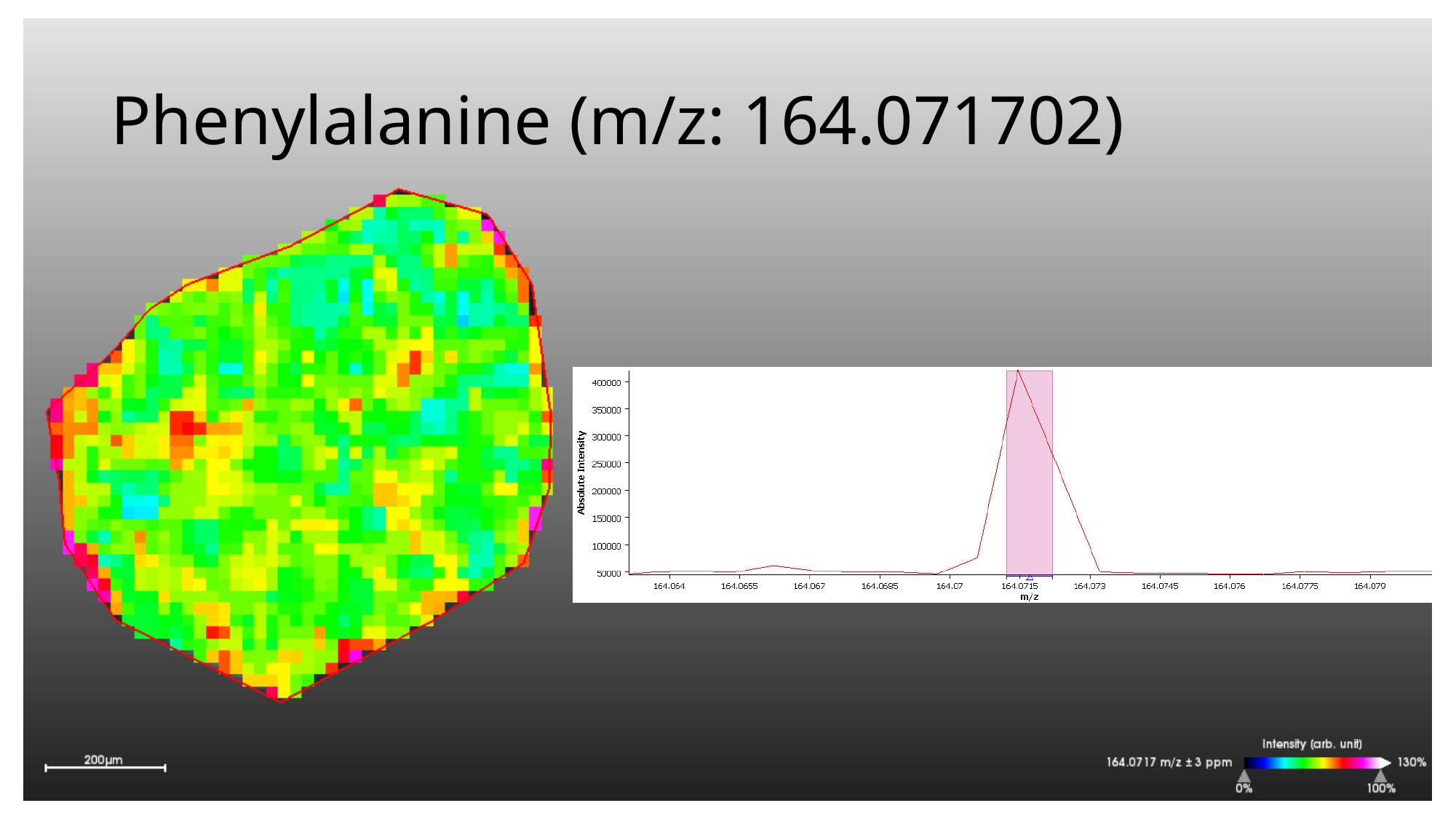

# Phenylalanine (m/z: 164.071702)

## Slide 16
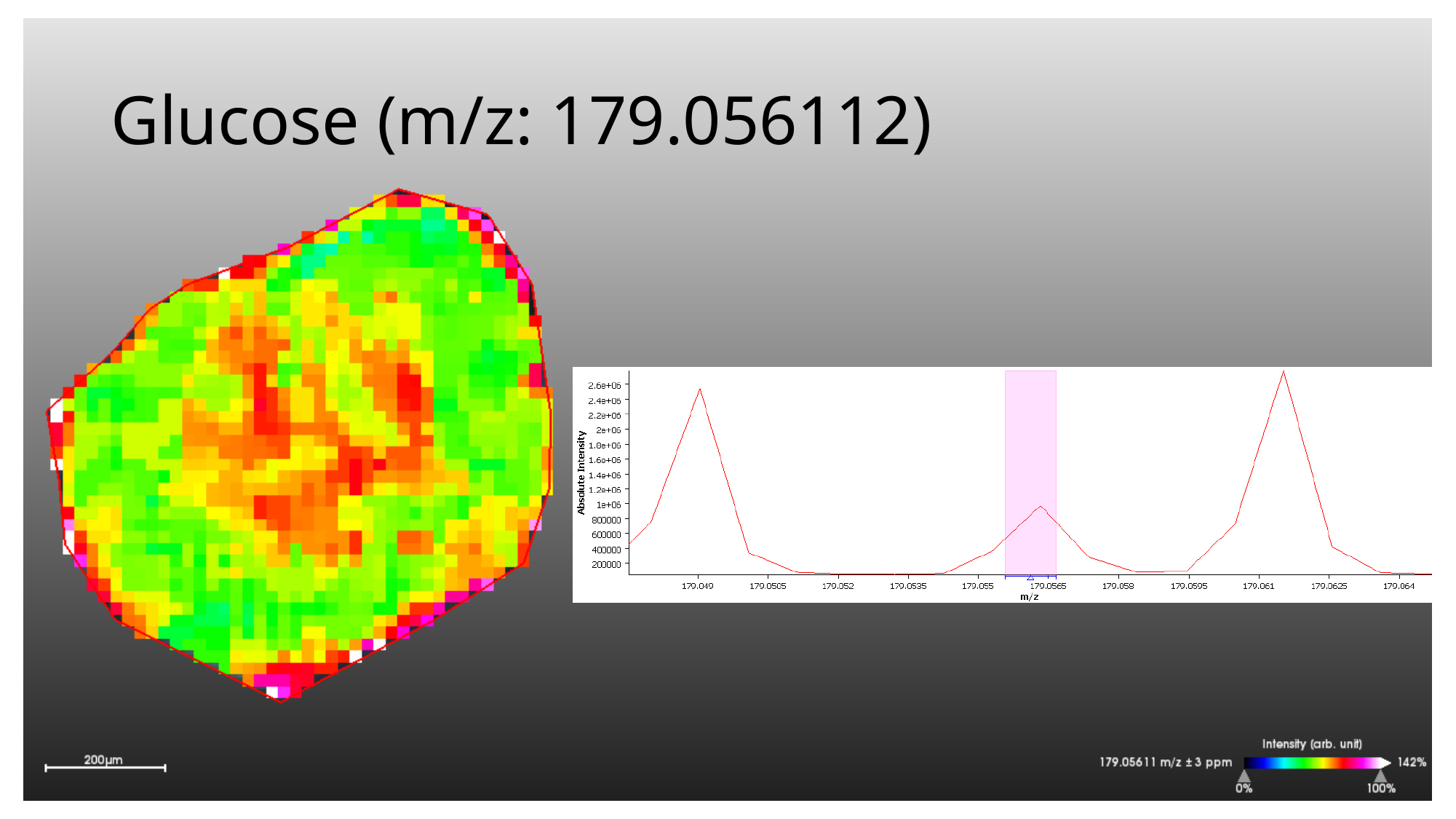

# Glucose (m/z: 179.056112)
